# Raman and AFM-IR Spectroscopy in the Characterization of Giant Plasma Membrane Vesicles Isolated from Microglia and Glioblastoma Cells

**DOI:** 10.1021/acs.langmuir.6c02670

**Published:** 2026-09-07

**Authors:** Jagoda Orleańska-Pietras, Katarzyna Pogoda

**Affiliations:** Institute of Nuclear Physics Polish Academy of Sciences, Krakow PL-31342, Poland

## Abstract

Giant plasma membrane vesicles (GPMVs) are widely used as cell-derived models of biological membranes, yet their spectroscopic characterization remains limited. In this work, we propose a preparation and analytical workflow enabling Raman and Atomic Force Microscopy-based infrared (AFM-IR) spectroscopic investigation of GPMVs isolated from microglial (human microglial clone 3, HMC3) and glioblastoma (LN229, U87 MG, and U251 MG) cell lines. Storage conditions and isolation parameters were systematically evaluated to ensure preservation of vesicle morphology and biochemical stability prior to spectroscopic measurements. Raman spectroscopy provided label-free molecular fingerprints of GPMV composition, revealing variations in lipid and protein contributions depending on the cellular origin of the vesicles. Complementary characterization was achieved using AFM-IR spectroscopy, enabling the detection of differences in carbohydrate-, protein-, and lipid-related vibrational bands at the nanoscale, without the need for sample labeling. Chemometric analysis, including principal component analysis and partial least squares discriminant analysis, further confirmed the spectroscopic discrimination of vesicles derived from different cell lines. To our knowledge, this work represents one of the first combined Raman and AFM-IR spectroscopic investigations of GPMVs, demonstrating the potential of multimodal vibrational spectroscopy for the characterization of cell-derived membrane systems. The presented methodology establishes a foundation for future spectroscopic studies of membrane-derived vesicles and their biochemical heterogeneity.

## Graphical abstract

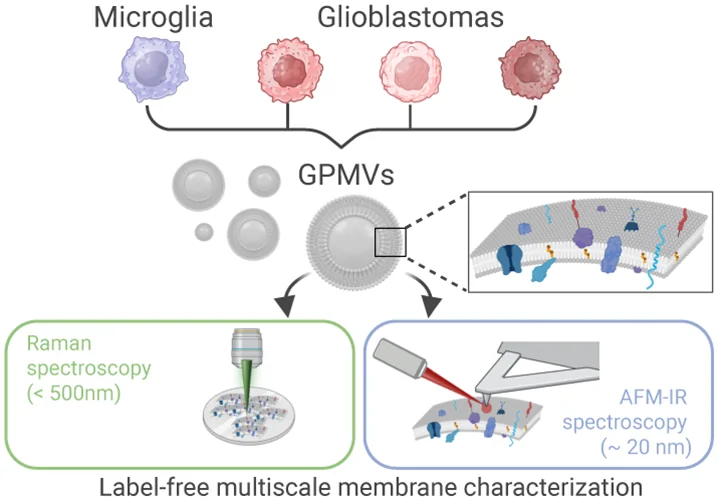

## Introduction

Giant plasma membrane vesicles (GPMVs) represent one of the most widely used model systems for investigating the structure and organization of cellular membranes.^1,2^ The cellular membrane, described by the fluid-mosaic model, consists of a complex and dynamic organization of lipids and proteins governed by thermodynamic constraints.^3^ Key components of this model include phospholipids, integral membrane proteins, and lipoproteins, which together constitute the fundamental architecture of biological membranes. As naturally cell-derived vesicles, GPMVs closely reflect and mimic the compositional complexity of the plasma membrane.^1,4^ In addition to membrane lipids and proteins, GPMVs retain cytoplasmic content (excluding intracellular organelles)^5^ and glycans,^6^ which occur in the form of glycoproteins and glycolipids forming the glycocalyx layer observed on the surface of cellular membranes and preserved in the generated vesicles.^2,7,8^ Importantly, GPMVs can be generated relatively easily and provide a stable model system for membrane studies without interference from the cellular environment, owing to the absence of the cortical actin cytoskeleton and the lack of ongoing cellular activity.^2,4^ Giant plasma membrane vesicles (GPMVs) are generated by chemically inducing plasma membrane blebbing in living cells. Vesiculation is typically achieved by treating cells with paraformaldehyde (PFA) and dithiothreitol (DTT). PFA cross-links membrane-associated proteins, whereas DTT reduces disulfide bonds. Together, these reagents weaken the coupling between the plasma membrane and the cortical cytoskeleton, promoting the formation and release of plasma membrane blebs. As a result, GPMVs retain much of the native lipid and protein composition of the plasma membrane while lacking intracellular organelles and an intact cytoskeleton, making them a valuable model system for investigating membrane structure and composition. The diameter of GPMVs typically ranges from approximately 3 to 20 μm.^9,10^

As a plasma membrane model GPMVs provide a suitable system for preparation, visualization, and investigation using a variety of microscopy and spectroscopy techniques.^11^ However, most studies on GPMVs have focused primarily on their biophysical properties. For example, parameters such as bending rigidity and membrane microviscosity have been investigated, both of which depend on the composition of the plasma membrane and the cellular origin of the vesicles.^12^ In addition, calculations of spontaneous membrane curvature have also been reported.^13^ But, the majority of GPMV research frequently involves the analysis of isolated membrane blebs using fluorescence microscopy combined with specific fluorescent probes.^14^ The membrane dye 3,3′-dilinoleyloxacarbocyanine perchlorate (DiO) has been used to stain the plasma membrane of cells and subsequently visualize the membrane in generated GPMVs. Immunostaining approaches, such as labeling with aquaporin-3, have also been applied to investigate membrane rupture during bleb formation and to develop functional GPMV platforms for simplified analysis of membrane properties.^15^ Additionally, methods have been developed to prepare planar GPMV samples using fluorescent probes such as octadecyl rhodamine B chloride, enabling further characterization by atomic force microscopy (AFM), although this approach remains experimentally challenging.^14,16^ Fluorescence-based investigations of GPMVs commonly employ membrane-sensitive dyes such as C-Laurdan^12,17^ and Rhodamine-DOPE.^18^ Fluorescent probes have also been used to study vesicle permeability and leakiness, for example using Alexa Fluor 647-ATP, fluorescein-labeled dextran, or 3-kDa dextran, to evaluate the porosity of GPMVs and their suitability as plasma membrane permeability models.^19^

Beyond fluorescence imaging, advanced microscopy techniques have also been applied to investigate membrane organization in GPMVs. These include super-resolution stimulated emission depletion microscopy combined with fluorescence correlation spectroscopy (STED-FCS), which has been used to study the role of the actin cortex and to examine lipid and protein dynamics in plasma membranes at the nanoscale.^20^ Laser scanning confocal microscopy and two-photon microscopy have also been employed for visualization and structural analysis of GPMVs.^5^

In contrast, relatively few studies have explored the application of vibrational spectroscopy for the characterization of GPMVs. Reported examples include surface-enhanced Raman spectroscopy (SERS), which has been used to investigate the binding of cholera toxin B subunits to GPMV membranes deposited on gold substrates in planar configurations, with spectral analysis supported by convolutional neural networks.^21^ Additionally, Fourier-transform infrared (FT-IR) spectroscopy has been applied to analyze lipid-derived signals from isolated membrane blebs under different temperature conditions, in combination with AFM and fluorescence microscopy.^22^

In this work, we present the application of Raman and Atomic Force Microscopy-infrared (AFM-IR) spectroscopy combined with chemometric analysis to detect and characterize differences between GPMVs isolated from selected microglial and glioblastoma cell lines. Raman spectroscopy and AFM-IR are label-free and nondestructive techniques that enable the investigation of biological samples without altering their biochemical composition. Application of AFM-based IR spectroscopy, provides chemical information with an approximate spatial resolution of 20 nm being primarily determined by the size of the AFM probe tip. It has become a powerful tool for nanoscale chemical characterization and has been successfully applied to the investigation of polymer nanostructures and nanocomposites,^23^ nanoplastics,^24^ polymer-based drug delivery systems,^25^ protein fibril organization,^26^ extracellular vesicles and lipid bilayers,^27^ microorganisms such as yeast,^28^ and cancer cells.^29^ Although Raman spectroscopy and AFM-IR provide consistent biochemical insights, they probe GPMVs at different spatial scales and therefore offer complementary analytical capabilities. Raman spectroscopy provides an overview of the overall molecular composition of GPMVs, whereas AFM-IR enables nanoscale chemical characterization of individual membrane regions, allowing the identification of local biochemical heterogeneity and chemically distinct regions. The combination of these two label-free, nondestructive techniques provides a comprehensive characterization of GPMVs while preserving their native biochemical composition and eliminating the need for additional sample modification.

This study also includes optimization of GPMV sample preparation without the use of additional preservatives, as well as evaluation of vesicle acquisition and storage conditions using spectroscopic monitoring. The obtained Raman and AFM-IR spectra were analyzed to assign characteristic vibrational signals associated with the biochemical components of individual GPMV populations and to identify compositional differences between vesicles derived from different cell lines—microglial and glioblastoma. The selected cell lines represent both normal and malignant brain cells. HMC3 microglial cells were used as a non-tumor reference, whereas LN229, U87, and U251 represent widely used glioblastoma models that reflect the biological heterogeneity of this highly aggressive brain tumor. Since glioblastoma progression and the development of drug resistance are closely associated with alterations in plasma membrane composition, GPMVs derived from these cell lines constitute a relevant model for investigating membrane-specific biochemical differences using label-free vibrational spectroscopic techniques. The proposed methodology for GPMV sample preparation, storage, and spectroscopic characterization, supported by statistical and chemometric analysis, provides a robust framework for future analytical studies of GPMVs and for advancing the spectroscopic investigation of plasma membrane.

## Materials and Methods

### Sample Preparation for Spectroscopic Measurements

GPMVs were isolated from four human cell lines: HMC3 (microglial cells, ATCC, Manassas, VA, USA) and three glioblastoma cell lines: LN229 (LN229, ATCC, Manassas, VA, USA), U87 MG (U87, ATCC, Manassas, VA, USA), and U251 MG (U251, ECACC 09063001, Merck, Darmstadt, Germany). Cells were seeded in T75 cell culture flasks (75 cm^2^ growth area) and cultured in appropriate media. LN229 cells were grown in DMEM (ATCC, Manassas, VA, USA) supplemented with 5% fetal bovine serum (FBS) and 1% penicillin–streptomycin solution. HMC3, U87, and U251 cells were cultured in EMEM medium (ATCC, Manassas, VA, USA) supplemented with 10% FBS and 1% penicillin–streptomycin solution. Cells were maintained in an incubator at 37 °C under a humidified atmosphere containing 5% CO_2_ until the desired confluence was reached.

GPMVs were generated by incubating cells for 3 h with an isolation buffer prepared according to the procedure described previously, with application of DTT/PFA reagents.^17^ After incubation, the supernatant containing the isolated membrane blebs was collected into 15 mL Falcon tubes and centrifuged. The resulting supernatant was transferred to new tubes and stored at 4 °C. After 7 days of storage in the isolation buffer, 50 μL of the vesicle suspension was collected from the bottom of the tubes and deposited onto calcium fluoride (CaF_2_) substrates (Φ 13 mm × 2 mm, Crystran Ltd., Poole, UK). Samples were kept under a fume hood for 30 min. Subsequently, approximately 30 μL of residual liquid was removed from the sample surface and drying was continued for an additional 30 min. The samples were then rinsed twice with ultrapure water to minimize potential spectral contributions from the isolation buffer. Finally, the samples were dried under a stream of nitrogen and stored in a desiccator until spectroscopic measurements were performed. For Raman and AFM-IR measurements, GPMVs were analyzed after deposition and drying on CaF_2_ windows. Due to the drying process, the vesicles were randomly distributed across the substrate, with the highest concentration typically observed near the periphery of the dried droplet. Spectroscopic measurements were therefore performed in these regions. After drying, GPMVs exhibited heterogeneous morphologies, including intact spherical vesicles and ruptured membrane deposits formed during the washing and drying procedures. These deposits typically formed layers with thicknesses of approximately 100–500 nm, which were suitable for both AFM-IR and Raman spectroscopic measurements.

### Optimization of GPMV Storage and Production

The stability of isolated GPMVs in the isolation buffer was evaluated by monitoring vesicle morphology during storage and by assessing the efficiency of vesicle production. Observations were performed using an inverted microscope Leica DMi8 (Leica Microsystems, Wetzlar, Germany) equipped with a 40× air objective (NA = 0.61, Leica Microsystems, Wetzlar, Germany). Micrographs were acquired in phase-contrast mode to visualize the structure of GPMVs stored under different temperature conditions: +4 °C, −20 °C, −80 °C, and liquid nitrogen (LN). In parallel, the production efficiency of GPMVs was evaluated separately for each investigated cell line. Quantitative analysis was performed by counting the number of vesicles and measuring their diameters from the recorded micrographs using ImageJ software. For each sample, five independent images of GPMVs deposited in a 96-well plate were analyzed. The average number of generated blebs was calculated and presented using linear plots. Vesicle diameter distributions were visualized using box–whisker plots (box: mean value and standard deviation, ±SD; whiskers: minimum and maximum values) for each sample. Graphs were generated using OriginPro software.

### Raman Spectroscopy

Raman measurements of GPMV samples were performed on CaF_2_ substrates using an inVia Raman spectrometer (Renishaw, UK) equipped with a CCD detector and coupled to an optical confocal microscope. Spectra were acquired using a 100× air objective (NA = 0.75, Leica Microsystems, Wetzlar, Germany). Raman spectra were recorded using a 532 nm laser line, with 75% laser power. The exposure time was set to 2 s with 10 accumulations per spectrum. Spectra were collected within the spectral range of 100–3200 cm^–1^. Approximately 110 individual Raman spectra were acquired for each sample. Following quality assessment, HMC3 (n = 95), LN229 (n = 104), U87 (n = 108), and U251 (n = 94) spectra were retained and included in the subsequent analyses. Spectra not included in the analysis were excluded because they had insufficient signal quality.

### AFM-IR Spectroscopy

AFM-IR measurements were performed using a NanoIR2 instrument (Bruker, Santa Barbara, CA, USA) equipped with a quantum cascade laser (QCL) system (MIRcat-QT, Daylight Solutions) consisting of four tunable laser chips that generate the infrared radiation source. Infrared spectra were collected with 3% laser power in the fingerprint region (764–1858 cm^–1^) and 20% laser power in the high-wavenumber region (2600–2996 cm^–1^). The spectral resolution was set to 2 cm^–1^. GPMVs were analyzed in contact mode based on AFM topography images acquired prior to spectroscopic measurements. Silicon gold-coated probes dedicated for AFM-IR measurements (PR-EX-nIR2-10, Bruker) were used, with a nominal resonance frequency of 13 ± 4 kHz and a spring constant of 0.07–0.4 N·m^–1^. All the measurements were performed in resonance-enhanced mode by matching the QCL repetition rate to the contact resonance frequency of the cantilever (approximately 180 kHz). A phase-locked loop (PLL) was used to track small resonance shifts during the measurements, with the PLL configured to a center frequency of 180 kHz and a tracking window of 50 kHz. AFM topography images were recorded at a scan rate of 0.5 Hz with a scan area of 20 μm × 20 μm and a resolution of 300 × 300 points. Approximately 60 individual AFM-IR spectra were collected for each sample. Measurements were performed by acquiring AFM topographies from three independently selected sample areas, followed by the collection of approximately 20 spectra from selected locations within each imaged area. Representative AFM topography is provided in the Supporting Information (Figure S1). Before each sample measurement, a background spectrum was acquired under identical experimental conditions and was automatically used by the instrument software to normalize the AFM-IR spectra, compensating for the wavelength-dependent output power of the quantum cascade laser (QCL). Approximately 80 individual AFM-IR spectra were acquired for each sample, of which HMC3 (n = 71), LN229 (n = 53), U87 (n = 74), and U251 (n = 56) were retained for subsequent analysis. Spectra not included in the analysis were excluded because they had insufficient signal quality.

### Spatial Resolution of Raman vs AFM-IR Measurements

The theoretical lateral spatial resolution of the Raman measurements, calculated according to the Abbe diffraction criterion, was approximately 0.43 μm for the 532 nm excitation laser and a 0.75 NA objective. In comparison, AFM-IR provides a spatial resolution of approximately 20 nm, determined primarily by the AFM probe tip. Consequently, Raman spectroscopy provides an averaged molecular fingerprint over a larger sampling area, whereas AFM-IR enables localized nanoscale chemical characterization. The complementary spatial resolutions and different contrast mechanisms of the two techniques allow comprehensive characterization of GPMVs across multiple length scales.

### Data Analysis

Raman spectra were initially preprocessed using WiRE 5.3 software (Renishaw, UK) to remove cosmic ray artifacts and subsequently exported to OPUS 7.2 software (Bruker Optik GmbH, Ettlingen, Germany). The spectra were truncated to the spectral range of 400–3100 cm^–1^ for further analysis.

AFM-IR spectra were first processed using Analysis Studio 3.14 software. Prior to each measurement, a background spectrum was acquired under identical experimental conditions and automatically subtracted from the measured spectra by the instrument software. Spectral smoothing was performed using the Savitzky–Golay algorithm (third-order polynomial, 5 side points), after which the spectra were exported to OPUS 7.2 for further preprocessing. Vector normalization (built-in OPUS routine, scaling each spectrum to unit vector length) was applied in the fingerprint region (780–1800 cm^–1^). Second-derivative AFM-IR spectra were calculated using the Savitzky–Golay method (9 smoothing points) to improve the resolution of overlapping bands, facilitate more accurate band assignment, reduce baseline contributions, and enhance the detection of subtle spectral differences between the analyzed samples.

For visualization and quantitative analysis, Raman spectra were smoothed (Savitzky–Golay, 9 smoothing points), baseline corrected, and vector normalized over the 400–3100 cm^–1^ spectral range. Integral intensities of selected Raman bands were then calculated. For AFM-IR data, the integral intensities of selected bands were determined from the second-derivative spectra.

Statistical evaluation of the spectral data was performed using analysis of variance (ANOVA) followed by Tukey’s post hoc test. Prior to statistical analysis, the data distribution was assessed using the Shapiro–Wilk normality test. Statistical analysis and graphical visualization were performed using OriginPro software. Statistical significance was defined as *p < 0.05, **p < 0.01, and ***p < 0.001. Data are presented as box-and-whisker plots, where the box represents the mean ± standard deviation (SD), and the whiskers indicate the minimum and maximum values.

Chemometric analyses were performed to visualize and confirm spectral differences between the investigated GPMV samples. Principal Component Analysis (PCA) was carried out using The Unscrambler X software (Camo Software, Oslo, Norway). Prior to PCA, Raman spectra were preprocessed by Savitzky–Golay smoothing (third-order polynomial, 15 points), linear baseline correction, and vector normalization over the 500–1800 cm^–1^ spectral range. Second-derivative AFM-IR spectra were linearly baseline corrected and vector normalized over the 764–1800 cm^–1^ fingerprint region before PCA.

Partial Least Squares Discriminant Analysis (PLS-DA) was performed using Raman spectra to discriminate GPMVs derived from normal microglial cells (HMC3) and glioblastoma cell lines (LN229, U87, and U251). Before model construction, spectra were preprocessed using constant offset elimination, linear baseline correction, multiplicative scatter correction (MSC), and vector normalization over the 500–1800 cm^–1^ spectral range. Model performance was evaluated by cross-validation, and regression coefficient loadings were analyzed to identify the spectral features contributing to sample discrimination.

To improve the clarity and reproducibility of the data analysis, each preprocessing workflow has been summarized in Table S1 (Supplementary Information).

## Results and Discussion

### Storage of GPMVs and Sample Preparation

Considering the subsequent spectroscopic investigation of isolated GPMVs (blebs), no additional preservatives were used during sample preparation in order to avoid introducing extraneous spectral signals that could interfere with characteristic bands originating from the vesicles. Therefore, the first step involved verification of GPMV morphology as a function of storage temperature over time using phase-contrast microscopy.

Figure S2 (Supplementary Information) presents representative bright-field images of GPMVs derived from LN229 cells stored under different temperature conditions. After 7 days of storage, the typical vesicular morphology was no longer observed for samples stored at −20 °C, −80 °C, or in liquid nitrogen (LN), in contrast to the control GPMVs. The obtained microscopic images suggest that storage at temperatures lower than +4 °C leads to disruption of the initial vesicle structure, likely resulting in membrane damage and leakage of vesicle contents into the surrounding solution. This effect is evidenced by the presence of deposits visible in Figure S2. In contrast, storage at +4 °C preserved the vesicular morphology for up to 42 days, with GPMVs displaying structures comparable to those observed immediately after isolation.

To determine the optimal duration of the GPMV isolation process for microglial and glioblastoma cell lines, the number and diameter of vesicles were analyzed as a function of isolation time. Figure 1 presents the production profiles of GPMVs after 1, 3, 6, and 24 h of the isolation process. Similar trends were observed for all investigated cell lines, indicating that both the number and diameter of GPMVs increase with increasing isolation time. The number of vesicles increased significantly during the first hours of isolation (1–3 h) but reached a plateau after 6–24 h, consistent with previous observations reporting stabilization of vesicle production after approximately 4 h of isolation.^9^ This behavior may indicate completion of vesicle formation or depletion of cells exposed to the isolation buffer containing PFA, a commonly used fixation agent.^30,31^ The diameter of GPMVs also increased during the isolation process but eventually stabilized, suggesting the formation of vesicles within a characteristic size range. In addition to isolation time, vesicle production was influenced by the initial cell confluence and the specific cell line subjected to the isolation procedure. These observations confirm that isolation conditions should be optimized depending on the biological system and experimental requirements.^32^

**Figure 1:**
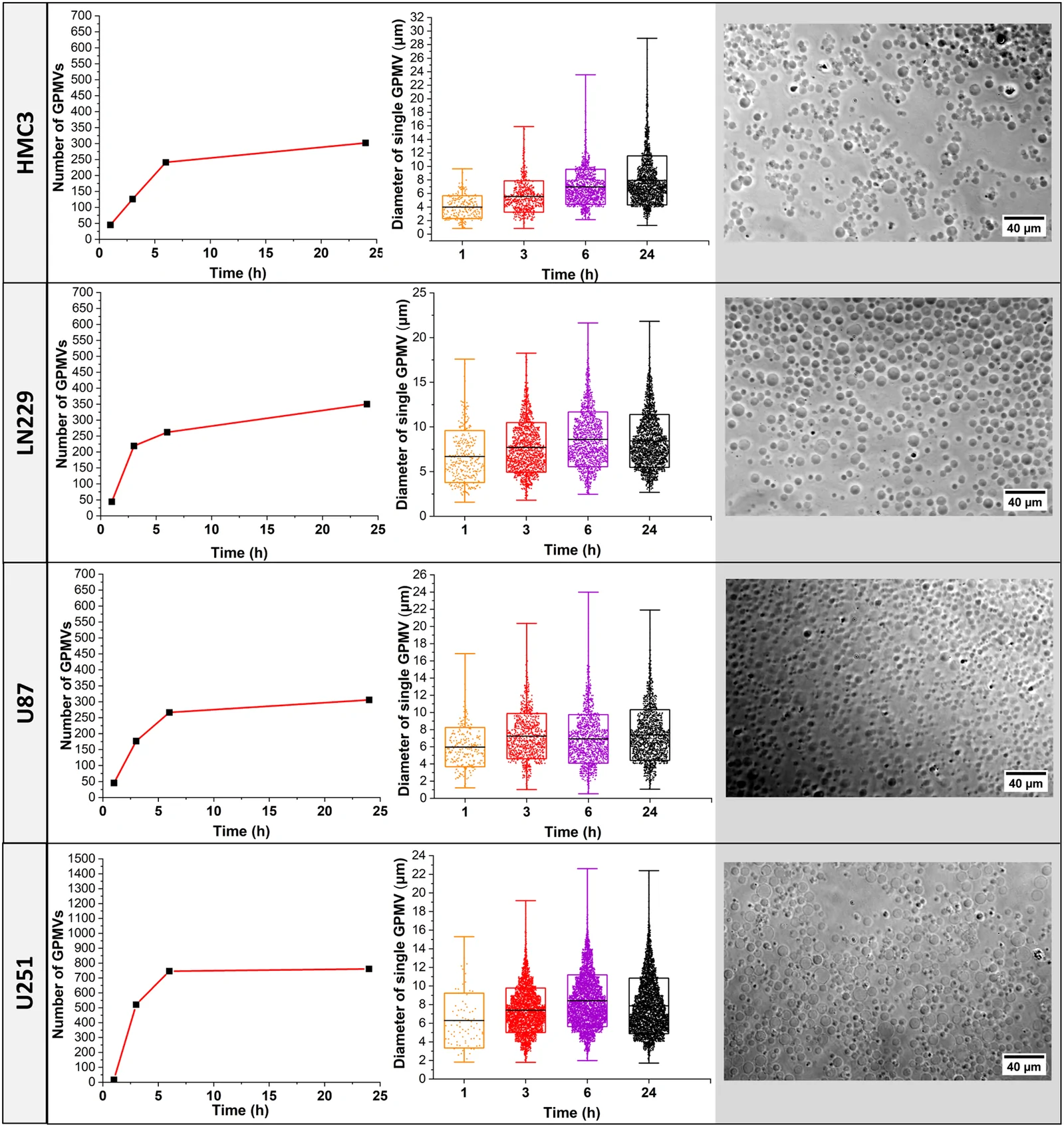
Linear plot showing the number of produced GPMVs as a function of isolation time (samples collected after 1, 3, 6, and 24 h from the initiation of isolation), together with box plots of GPMV diameters. The third-column shows representative bright-field images of blebs after 3 h of isolation in solution (an objective with 40× magnification was used). Cell confluence prior to isolation: HMC3—60%, LN229—60%, U87—60%, U251—80%.

During microscopic observations, differences in the spatial behavior of GPMVs derived from different cell lines were also noted (Figure 1). Vesicles originating from HMC3 cells tended to form clusters, whereas LN229-derived vesicles remained more dispersed, suggesting different surface properties of the vesicles. Furthermore, HMC3 cells produced fewer vesicles compared with the other investigated cell lines during the early stages of isolation. In contrast, U87 cells generated GPMVs more readily than the other cell lines, even at lower cell confluence. These observations indicate that vesicle production efficiency depends on the cellular origin of the GPMVs.

Based on these results, an isolation time of 3 h was selected as optimal for further experiments. This duration provided a sufficient number of vesicles while preserving their structural integrity, enabling reliable preparation of samples for subsequent spectroscopic measurements.

The unique morphology of isolated GPMVs and the possibility that the isolation buffer may influence vesicle stability and biochemical composition during storage at +4 °C necessitated evaluation of optimal storage conditions for the blebs after isolation. Raman spectroscopy was therefore applied to monitor potential biochemical changes occurring during storage of GPMVs in the isolation buffer. For spectroscopic measurements, dried vesicles were used because GPMVs tend to spread in a liquid buffer environment, which complicates reproducible spectral acquisition.

Figure 2A presents the average Raman spectra obtained for GPMVs isolated from the U87 cell line and stored in the isolation buffer at +4 °C for different periods of time. Analysis of the Raman spectra indicates gradual changes in the biochemical composition of the vesicles during storage. To quantify these potential modifications, ANOVA analysis (Figure 2B) was performed for selected Raman bands and band ratios: 720 cm^–1^ (lipids containing choline groups, characteristic of phospholipids),^33,34^ the ratio 1035/1005 cm^–1^ (associated with cross-linked protein structures),^35^ and the ratio 1660/1440 cm^–1^ (related to the degree of lipid unsaturation).^36–38^ To further evaluate the spectral changes associated with prolonged GPMV storage, PCA was performed by grouping Raman spectra into two time regimes: 1–14 days and 21–35 days (Figure 2D). The score plot revealed differentiation between the two groups, primarily along PC4 (6% of the total variance), while PC1 accounted for 50% of the overall spectral variance. Analysis of the corresponding PC4 loading identified the spectral features contributing to this differentiation. Positive loading features, associated predominantly with the 21–35-day group, were observed around 720, 856, 1005, 1090, 1130, 1340, 1440, and 1694 cm^–1^, whereas negative loading features, associated predominantly with the 1–14-day group, occurred around 640, 820, 995, 1020, 1206, 1215, 1405, 1544, 1605, and 1650 cm^–1^. As individual Raman bands may contain contributions from multiple molecular species, these loading features were interpreted collectively as changes in the overall spectral fingerprint rather than as markers of individual biochemical components. A detailed assignment of the Raman spectral features is provided in the Supplementary Information (Table S2). These results demonstrate that prolonged storage is accompanied by changes in the overall Raman spectral profile of the GPMV.

**Figure 2:**
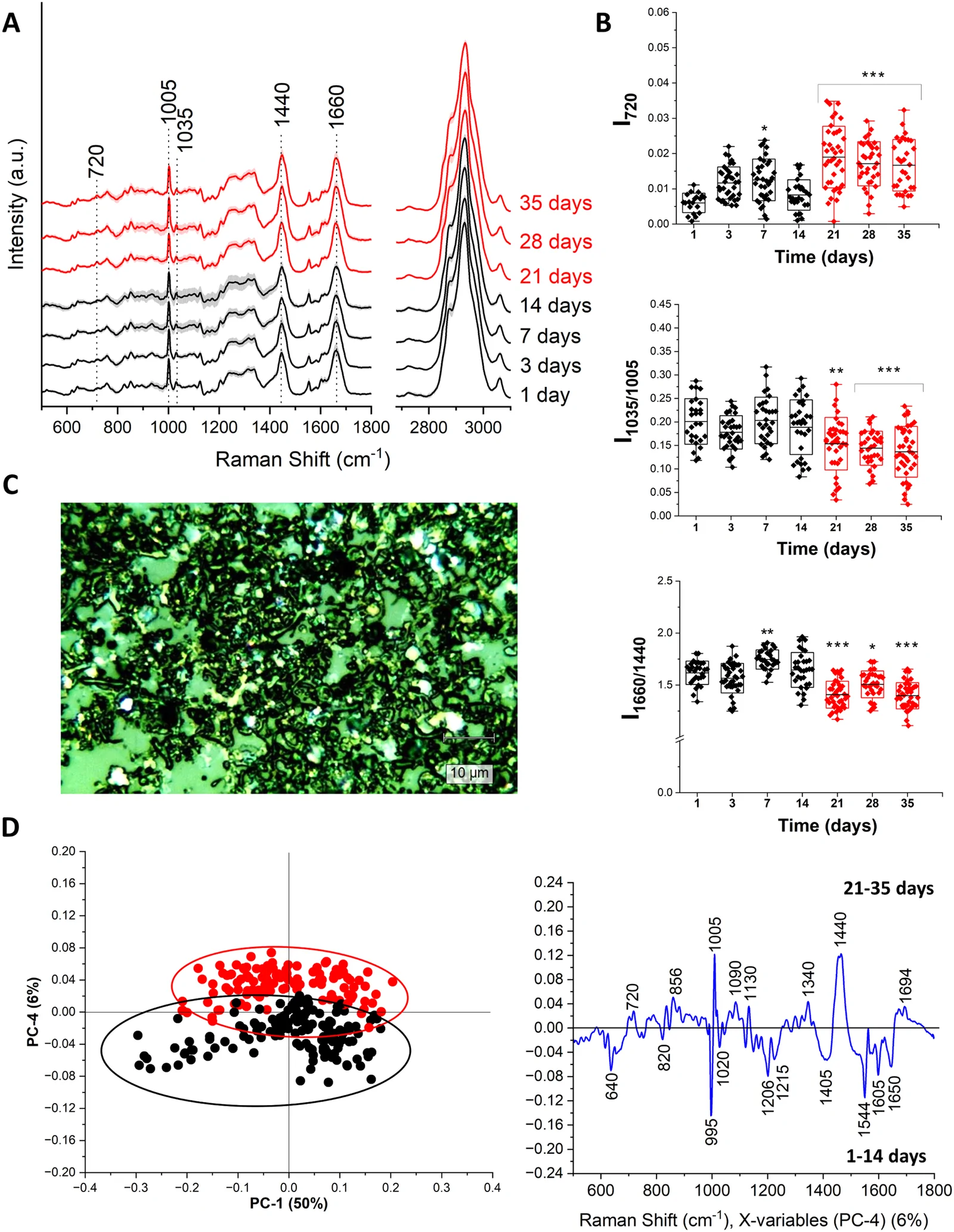
(A) Average Raman spectra and (B) ANOVA analysis of integral intensities of the Raman band at 720 cm^–1^ and band ratios 1035/1005 and 1660/1440 cm^–1^ for dry GPMVs derived from U87 cells (confluence 60%) during storage. (C) Representative micrograph showing accumulation of dried U87-derived blebs on a CaF_2_ substrate. (D) PCA results for 1–14 days (black), and 21–35 days (red) group of Raman spectra. *p < 0.05; **p < 0.01; ***p < 0.001; data without *—no statistical significance versus day 1 (day of isolation).

The obtained results indicate that noticeable spectral modifications appear after more than 14 days of storage in the isolation buffer at +4 °C. Within the first 14 days, no statistically significant differences in lipid or protein-related Raman signals were observed compared with vesicles analyzed immediately after isolation. These findings suggest that GPMVs can be stored for up to 14 days under refrigerated conditions without detectable alterations in their morphology (Figure 1) or biochemical composition (Figure 2, Figure S3).

Beyond this period, gradual changes in the biochemical composition of the vesicles become apparent. In particular, a decrease in the intensity of the C=C stretching band near 1660 cm^–1^ (expressed as the 1660/1440 cm^–1^ ratio) suggests possible lipid oxidation processes. Similar spectral changes have previously been reported for biological samples stored under conditions that promote oxidative degradation, including exposure to oxygen, temperature fluctuations, light, or prolonged storage in solution.^39–41^ Additionally, the presence of DTT and PFA in the isolation buffer may contribute to biochemical alterations of the vesicles during prolonged storage. Both reagents are known to interact with proteins and influence membrane-associated biomolecules during vesicle isolation procedures.^42,43^

### Analysis of GPMV Heterogeneity by Raman Spectroscopy

Further analysis was performed on dried GPMVs stored in the isolation buffer for 7 days after isolation. Raman spectroscopy was applied to investigate potential biochemical differences between vesicles derived from four selected cell lines: microglial HMC3 cells and glioblastoma cell lines LN229, U87, and U251. The average Raman spectra of GPMVs obtained from the investigated cell lines are presented in Figure 3A, together with the main characteristic bands, while detailed spectral assignments are summarized in Table S2 in the Supplementary Information.

**Figure 3:**
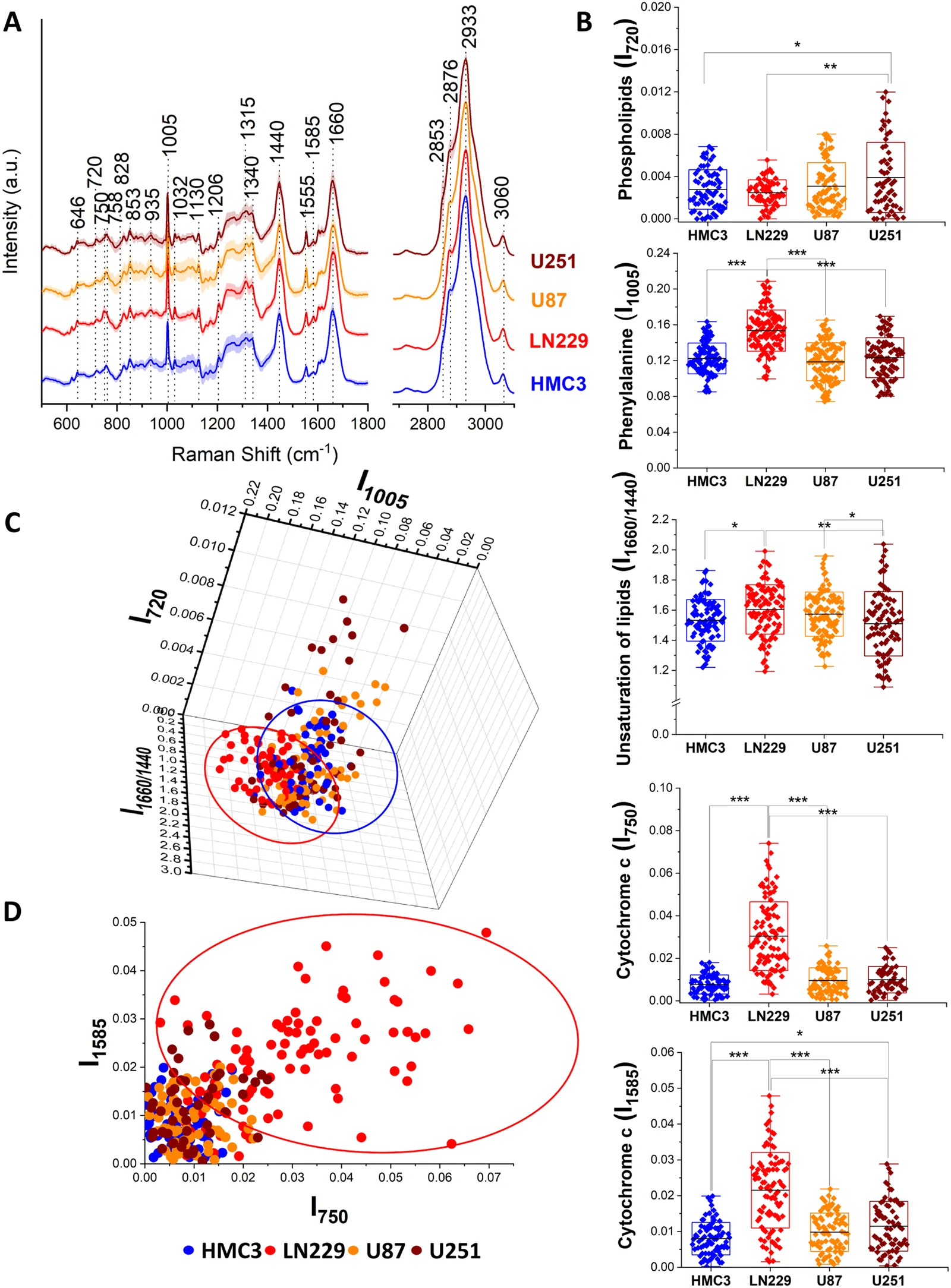
(A) Average Raman spectra of dry GPMVs isolated from HMC3, LN229, U87, and U251 cells. (B) ANOVA analysis of selected Raman band integral intensities. (C) Three-dimensional (3D) and (D) two-dimensional (2D) plots visualizing differences in Raman spectral features between GPMVs derived from the investigated cell lines.

The spectral profiles reveal noticeable differences primarily in protein- and lipid-related signals. Overall, the spectra are dominated by protein contributions, which is consistent with the high protein content typically associated with plasma membranes. Importantly, no characteristic signals originating from nucleic acids were observed, such as the Raman band near 785 cm^–1^. The absence of this band confirms the lack of DNA and RNA components within the isolated GPMVs,^44^ which is consistent with previous reports indicating that nucleic acids are not present inside GPMV structures.^42,45^

Semi-quantitative evaluation of Raman band intensities (Figure 3B) revealed variations in biochemical composition depending on the cellular origin of the vesicles. ANOVA analysis demonstrated that U251-derived vesicles exhibit a higher intensity of the band at 720 cm^–1^, associated with choline-containing phospholipids, compared with LN229 and HMC3 vesicles. In contrast, the phenylalanine band at 1005 cm^–1^ showed significantly higher intensity in vesicles derived from LN229 cells, indicating an increased contribution of protein-related components in these samples. Differences were also observed in lipid unsaturation levels. ANOVA analysis of the 1660/1440 cm^–1^ intensity ratio, commonly used as an indicator of lipid unsaturation^37,38,44,46^ suggests that LN229-derived vesicles contain a higher proportion of unsaturated lipids compared with HMC3 and U251 GPMVs. Additionally, statistical analysis revealed significant differences in lipid-related signals between vesicles derived from U87 and U251 cells. Notably, LN229-derived vesicles exhibit increased Raman intensity at 750 and 1585 cm^–1^ (Figure 3B), which are characteristic bands associated with porphyrin-containing proteins, including cytochrome-related structures present in cells.^47,48^ These spectral features suggest distinct biochemical characteristics of vesicles originating from this glioblastoma cell line.

To further visualize the observed spectral differences, the integral Raman band intensities were represented using two-dimensional and three-dimensional plots (Figure 3C,D). These visualizations clearly demonstrate the separation of the LN229 dataset from the remaining samples, indicating substantial differences in biochemical composition between vesicles derived from this cell line and those obtained from the other investigated cell lines.

Chemometric analysis further demonstrated differences in the Raman spectral profiles of GPMVs derived from the investigated cell lines (Figure 4). Principal Component Analysis (PCA) of the Raman spectra was performed using pairwise comparisons between GPMVs derived from HMC3 microglial cells and each glioblastoma cell line (Figure 4A). The score plots revealed varying degrees of differentiation between the investigated groups, while the corresponding loading plots identified the spectral variables contributing to these differences. For the HMC3 versus LN229 comparison, PC1 and PC3 accounted for 47% and 5% of the total variance, respectively. The PC3 loading indicated contributions from spectral features at 750, 1005, 1130, 1315, 1585, and 1672 cm^–1^, predominantly associated with protein-related vibrations, including contributions from porphyrin-containing proteins. For the HMC3 versus U87 comparison, PC1 and PC3 accounted for 59% and 10% of the variance, respectively. Spectral features contributing to the differentiation included protein-associated bands at 779, 864, 942, 1340, and 1555 cm^–1^, together with bands at 1090, 1130, 1397, and 1460 cm^–1^ that contain contributions from lipid-related vibrations. In the HMC3 versus U251 comparison, PC1 and PC3 accounted for 34% and 11% of the variance, respectively, with contributions from several protein-associated spectral features, including those at 1145, 1294, 1378, 1486, and 1555 cm^–1^. Spectral features around 779, 801, and 1044 cm^–1^ were also observed in this comparison. Since some of these bands overlap with those detected in the isolation-buffer control, we cannot exclude the possibility that residual buffer components may contribute to the Raman spectra and complicate the interpretation of the HMC3 versus U251 comparison. The Raman spectrum of the dry isolation buffer crystals is provided in the Supplementary Information (Figure S4).

**Figure 4:**
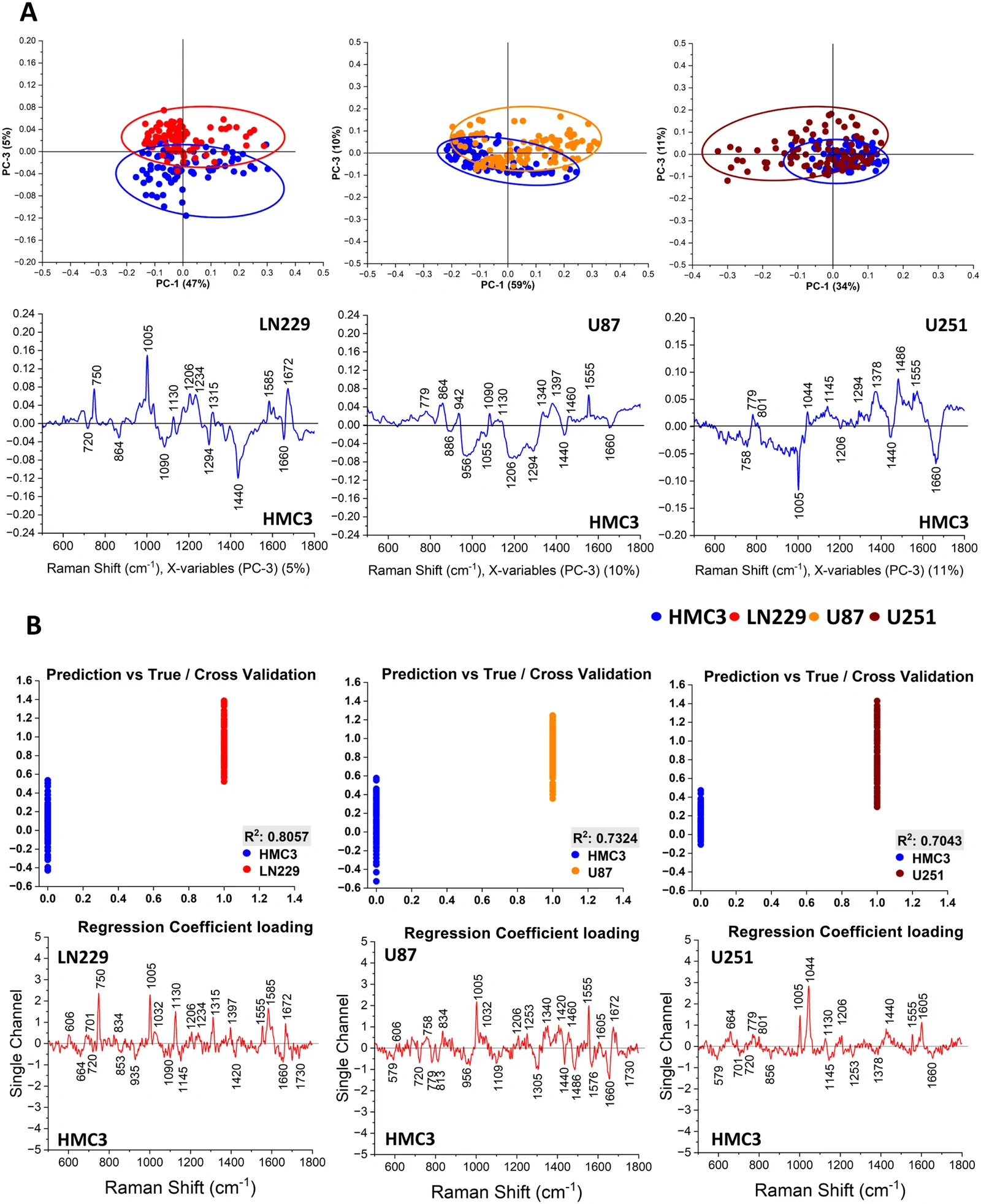
Chemometric analysis of Raman spectra of dry GPMVs: (A) principal component analysis (PCA) and (B) partial least squares discriminant analysis (PLS-DA) performed in the spectral region 500–1800 cm^–1^.

Because individual Raman bands may contain contributions from multiple molecular species, the PCA loadings were interpreted collectively as spectral fingerprints rather than as exclusive markers of individual biochemical components. Nevertheless, several recurrent spectral features contributed to the pairwise comparisons. In particular, the regions around 1440 and 1660 cm^–1^ contributed repeatedly to the characterization of HMC3-derived GPMVs. In the HMC3 versus LN229 comparison, contributions from the phospholipid-associated bands around 720 and 1090 cm^–1^ were also observed for HMC3-derived GPMVs. A detailed assignment of the Raman spectral features is provided in the Supplementary Information (Table S2).

Bands such as 1440, 1660 cm^–1^, repeatedly contributed to the characterization of HMC3-derived vesicles. Additionally, for the analysis performed between HMC3 and LN229, vesicles derived from microglial cells show an increased phospholipid content (720, 1090 cm^–1^). The remaining bands recorded in the analyses, such as 758, 864, 886, 956, 1005, 1055, 1206, 1294 cm^–1^ obtained for HMC3 vesicles, are bands derived from proteins, which suggests that differences between the analyzed vesicles occur mainly in the context of protein content.

To further evaluate the spectral differences between GPMVs of different cellular origins, Partial Least Squares Discriminant Analysis (PLS-DA) was applied to the Raman dataset^49^ (Figure 4B and Figure S5). Pairwise PLS-DA models were constructed to compare HMC3-derived GPMVs with each glioblastoma-derived population (Figure 4B), while additional models using LN229 as the reference were used to compare LN229 with HMC3, U87, and U251 (Figure S5). Cross-validation of the latter models yielded R^2^ values of 0.8135, 0.8273, and 0.8333 for LN229 versus HMC3, U87, and U251, respectively, supporting reproducible spectral differentiation between these GPMV populations. Analysis of the regression coefficient loadings revealed that the differentiation of LN229-derived GPMVs involved several spectral regions associated predominantly with protein-related vibrations. In particular, contributions were observed around 750, 1005, 1130, 1315, 1585, and 1672 cm^–1^, including spectral features associated with porphyrin-containing proteins, phenylalanine, and the amide I region. Lipid-associated spectral features, including the regions around 701, 720, 1090, 1420, and 1730 cm^–1^, also contributed to individual pairwise comparisons. Importantly, these loading features represent spectral variables contributing to discrimination between the compared groups and should not be interpreted as exclusive biochemical markers of individual cell lines. For comparisons involving U251-derived GPMVs, interpretation of features around 779–801 and 1044 cm^–1^ requires additional caution because these regions overlap with bands observed in the isolation-buffer control. Residual buffer components may therefore partially contribute to the Raman-based discrimination of this group. Overall, the pairwise PCA and PLS-DA analyses demonstrate differences in the Raman spectral fingerprints of GPMVs according to their cellular origin, with the PLS-DA results additionally indicating that LN229-derived GPMVs exhibit spectral characteristics that distinguish them from both microglial HMC3-derived GPMVs and GPMVs derived from the other glioblastoma cell lines.

### Application of AFM-IR Spectroscopy to GPMV Biochemical Characterization

AFM-IR spectroscopy was applied to complement the Raman results and to obtain local information about the biochemical composition of plasma membrane vesicles derived from microglial and glioblastoma cells. Statistical evaluation of selected infrared bands using ANOVA revealed significant differences in band intensities among vesicles originating from the investigated cell lines. Figure 5A and Figure 5B present the average AFM-IR spectra and their second-derivative representations for the analyzed GPMVs, respectively. The highlighted bands, described in detail at Supplementary Information (Table S3) correspond to vibrations associated with carbohydrates, proteins, and lipids, with detailed assignments provided in the Supplementary Information. Semi-quantitative comparison of selected bands is shown in Figure 5C. Infrared bands characteristic of carbohydrates and polysaccharides (1054 and 1154 cm^–1^) showed a significantly higher contribution in microglial (HMC3) vesicles compared with those derived from glioblastoma cells. In contrast, the lowest intensities of these bands were observed for LN229 vesicles, while no statistically significant differences were detected between U87 and U251 samples. Protein-related signals were evaluated using the amide I and amide II bands. The highest total contribution of these bands was observed for U251 vesicles, whereas HMC3 vesicles exhibited lower overall amide band intensities. Analysis of the secondary protein structure, expressed as the β-sheet/α-helix ratio (1694/1664 cm^–1^),^38^ showed the lowest values for U251 samples. No significant differences in this parameter were observed between HMC3 and U87 vesicles. Lipid-related bands at 1744 and 1246 cm^–1^ and the ratio 2852/2958 cm^–1^, associated with fatty acid chain length,^50^ also revealed significant biochemical differences between vesicles derived from the investigated cell lines. In particular, HMC3 vesicles exhibited the highest contribution of cholesteryl ester-related signals, in contrast to Raman results, which suggested significant contribution of cholesteryl esters for LN229 samples. The difference might be related to AFM-IR measurements in nanometer scale (the AFM tip apex radius on the order of 20 nm), what contributes to receiving spectra that characterize sample at the very local level. On the other hand, U251 vesicles showed an increased contribution of phospholipid-related bands, together with longer fatty acyl chains. The lowest values of the fatty acyl chain length parameter were observed for vesicles derived from U87 cells.

**Figure 5:**
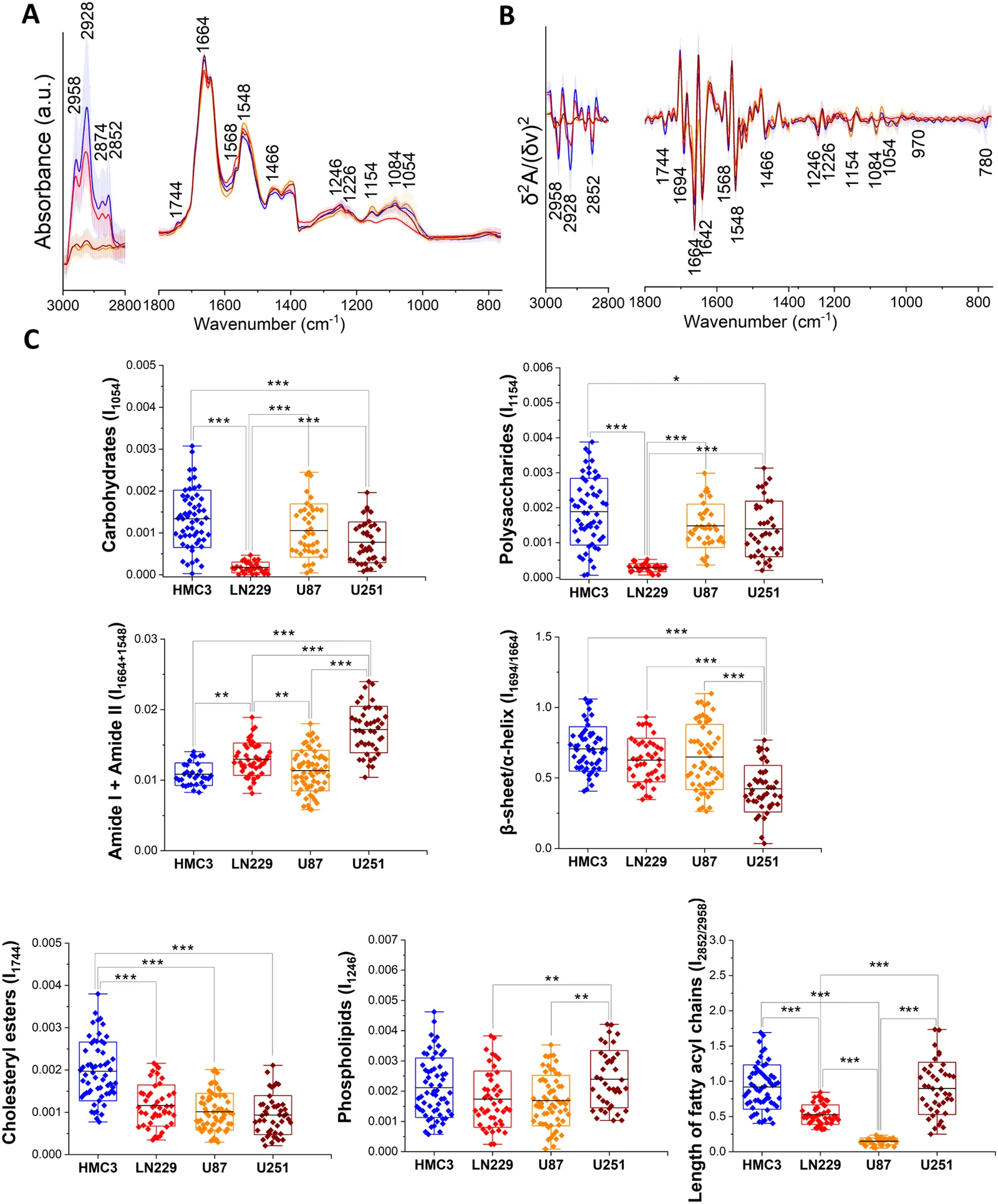
(A) Average AFM-IR spectra and (B) second-derivative AFM-IR spectra of dry GPMVs. (C) ANOVA analysis of selected integral intensities of IR bands.

Chemometric analysis of the AFM-IR spectra was performed for the investigated GPMV samples using pairwise comparisons between GPMVs derived from HMC3 microglial cells and those derived from each glioblastoma cell line separately (Figure 6). This approach allowed the spectral variables contributing to the differentiation of each pair of GPMV populations to be evaluated independently. For the HMC3 versus LN229 comparison, PC1 and PC4 accounted for 21% and 7% of the total variance, respectively, with differentiation between the groups observed primarily along PC4. Analysis of the corresponding PC4 loading revealed contributions from protein-associated bands at 1634 cm^–1^ (amide I, including β-sheet contributions), 1568 cm^–1^ (amide II), and 1514 cm^–1^ (tyrosine), as well as lipid- and phospholipid-associated features around 1456 and 1232 cm^–1^. The region around 1068 cm^–1^ contains contributions from carbohydrates, lipids, and glycolipids, whereas the feature around 790 cm^–1^ is associated with carbohydrate vibrations. For the HMC3 versus U87 comparison, PC1 and PC3 accounted for 25% and 8% of the total variance, respectively, with differentiation observed predominantly along PC3. The corresponding loading indicated contributions from protein-associated bands at 1642 cm^–1^ (amide I, including α-helical contributions) and 1530 cm^–1^ (amide II). Additional contributions were observed in lipid- and phospholipid-associated regions around 1232 and 1084 cm^–1^, while the bands around 1154 and 1026 cm^–1^ were associated predominantly with polysaccharide and lipid/sphingolipid vibrations, respectively. The corresponding PC1 loading is provided in the Supplementary Information (Figure S6A). For the HMC3 versus U251 comparison, PC1 and PC3 accounted for 22% and 9% of the total variance, respectively. The score plot showed partial differentiation between the groups, predominantly along PC3. The corresponding loading revealed contributions from the amide I region at 1684 and 1654 cm^–1^, associated with β-sheet and α-helical protein structures, respectively, together with the amide II band at 1558 cm^–1^. Additional contributions were observed around 1456 cm^–1^ (lipid/phospholipid vibrations), 1232 cm^–1^ (phospholipids), 1068 cm^–1^ (carbohydrates, lipids, and glycolipids), and 790 cm^–1^ (carbohydrates). The corresponding PC1 loading is presented in the Supplementary Information (Figure S6B).

**Figure 6:**
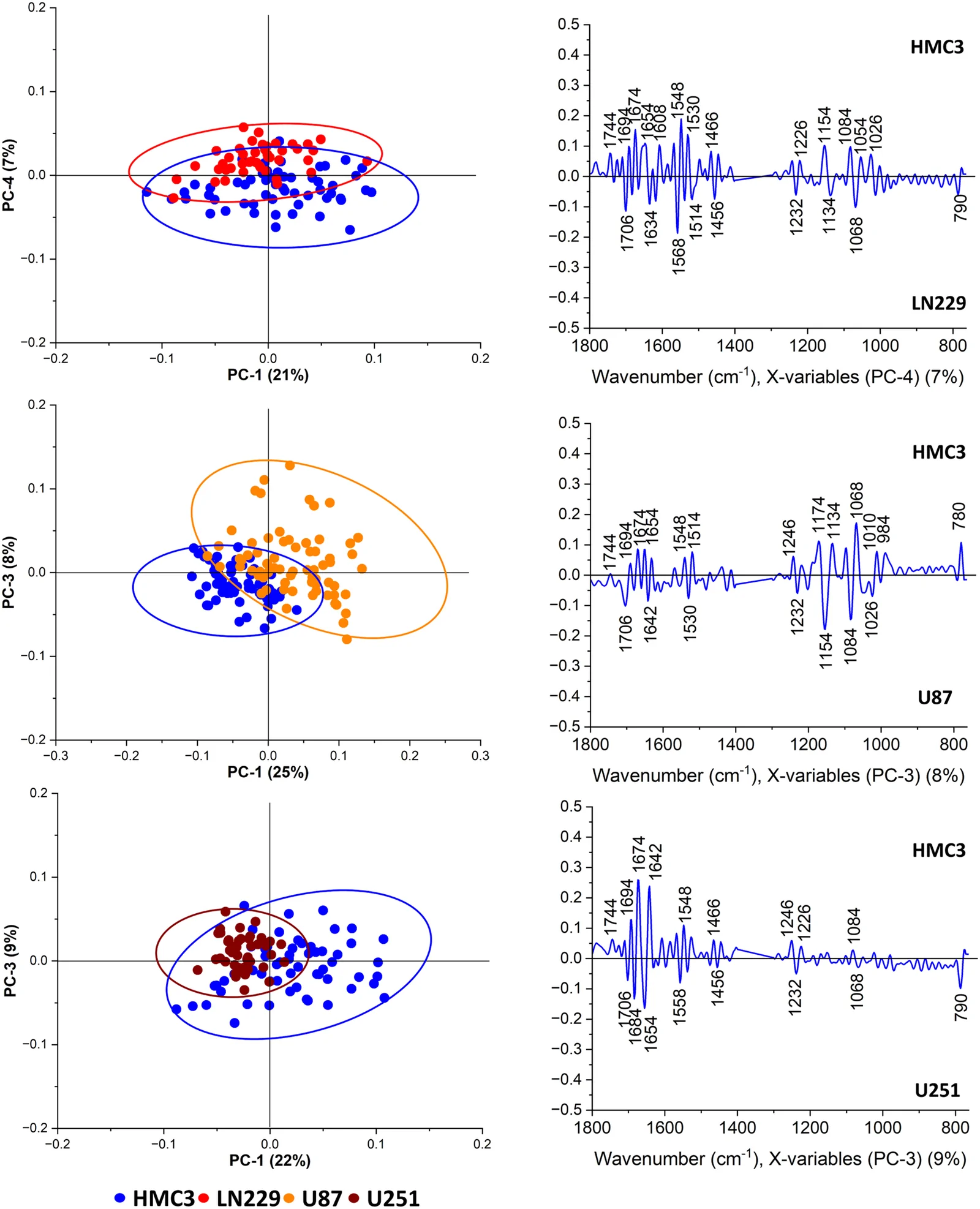
PCA of second-derivative AFM-IR spectra of GPMVs derived from microglial and glioblastoma cell lines. PCA score plots (left) and corresponding loading plots (right) are shown for pairwise comparisons of HMC3 versus LN229, HMC3 versus U87, and HMC3 versus U251. The analysis was performed in the spectral ranges of 1800–1400 and 1300–764 cm^–1^. The percentage of variance explained by each principal component is indicated on the corresponding axis.

Across the three pairwise comparisons, several spectral regions repeatedly contributed to the differentiation of HMC3-derived GPMVs from glioblastoma-derived GPMVs. In particular, recurrent contributions were observed in the amide I and amide II regions, including features around 1694, 1674, and 1548 cm^–1^, as well as in the lipid-associated carbonyl region around 1744 cm^–1^. The recurrent contribution of the amide I region suggests that differences in protein secondary structure contribute to the spectral distinction between microglial- and glioblastoma-derived GPMVs. However, because individual IR bands may contain contributions from multiple molecular species, the PCA loadings were interpreted collectively rather than as exclusive biochemical markers of individual GPMV populations. Detailed assignments of the AFM-IR bands are provided in the Supplementary Information (Table S3).

Although the dried isolation buffer exhibits strong AFM-IR bands at 1462, 1316, 1224, 1174, and 1042 cm^–1^ that overlap with biologically relevant spectral regions, additional control measurements demonstrated that measurable AFM-IR signals from the buffer were associated predominantly with visible crystalline deposits (Figure S7, Supplementary Information). Measurements performed on small buffer crystals yielded only weak spectral contributions, whereas no detectable AFM-IR signal was observed in regions between visible crystals (Figure S8, Supplementary Information). Since the GPMV samples were rinsed twice with ultrapure water prior to drying and AFM-IR spectra were acquired from regions without crystalline deposits, as verified by AFM topography, the contribution of residual isolation buffer to the analyzed AFM-IR spectra was considered negligible.

Overall, the AFM-IR chemometric analysis confirms and completes information about the biochemical differences detected by Raman spectroscopy and demonstrates that nanoscale infrared measurements provide complementary molecular information about lipid, protein, and carbohydrate distributions in GPMV-derived membranes isolated from different cellular origins.

Although Raman and infrared spectroscopy provide complementary biochemical information, they are governed by different physical principles and therefore differ in their sensitivity to specific molecular vibrations. In biological samples such as GPMVs, Raman spectra are dominated primarily by signals originating from proteins and lipids, whereas vibrations associated with carbohydrates and polysaccharides are generally weak and often overlap with stronger protein- and lipid-related bands.^44^ Subtle differences in carbohydrate content are difficult to resolve using Raman spectroscopy alone. In contrast, infrared spectroscopy is particularly sensitive to carbohydrate-related vibrations, making AFM-IR well suited for detecting differences in the polysaccharide- and carbohydrate-associated bands observed in the microglial and glioblastoma-derived GPMVs.^51^ This complementary sensitivity of Raman and AFM-IR is one of the principal motivations for combining both techniques in the present study. Consequently, application of AFM-IR may be used to study individual components of vesicle structures in the future.

## Conclusion

This study presents a spectroscopic workflow for the preparation and characterization of GPMVs derived from microglial and glioblastoma cell lines. The results establish practical guidelines for GPMV sample preparation and storage prior to spectroscopic measurements, demonstrating that vesicle morphology and biochemical stability are preserved for up to 14 days when stored at +4 °C in the isolation buffer. In contrast, storage at lower temperatures leads to disruption of vesicle structure, emphasizing the importance of controlled storage conditions for reliable spectroscopic analysis. Raman and AFM-IR spectroscopy as a complementary technique, enabled also label-free characterization of the biochemical composition of isolated vesicles and revealed measurable differences in lipid, protein, and carbohydrate contributions depending on the cellular origin of the GPMVs. Raman spectroscopy provided global molecular fingerprints of membrane composition, while AFM-IR spectroscopy enabled nanoscale infrared characterization of membrane deposits, offering complementary information about biochemical heterogeneity like biochemical information about secondary structure of proteins and about carbohydrates. Chemometric analysis further confirmed that spectroscopic signatures of GPMVs samples can discriminate vesicles deposits derived from different cell lines, highlighting variations in lipid organization, protein contributions, and membrane-associated components such as phospholipids and cholesterol-related species. The presented methodology demonstrates that GPMVs can serve as a robust plasma membrane model compatible with vibrational spectroscopic techniques. Importantly, the combination of Raman and AFM-IR spectroscopy provides a powerful analytical approach for investigating the molecular composition of cell-derived vesicles without the need for labeling or extensive sample modification. This approach may facilitate further investigations of plasma membrane organization, lipid–protein interactions, and biochemical alterations associated with cellular processes and pathological states.

## Supplementary Material



## Data Availability

The raw spectra supporting the findings of this study are publicly available in the RODBUK repository under 10.48733/IFJPAN/6DRQYU.

## Notes

Additional experimental details; materials and methods; photographs of the experimental setup and samples; supporting chemometric results (PDF)
