# Supplementary material for: Raman and AFM-IR Spectroscopy in the Characterization of Giant Plasma Membrane Vesicles Isolated from Microglia and Glioblastoma Cells
Source: Langmuir. 2026 Sep 7;42(37):27119–33. doi: 10.1021/acs.langmuir.6c02670 (PMC13602913; doi:10.1021/acs.langmuir.6c02670)
Supplement: Supplementary file 1 [file la6c02670_si_001.pdf]

Supplementary Information

Raman and AFM-IR spectroscopy in characterization of Giant Plasma Membrane Vesicles isolated from microglia and glioblastoma cells

Jagoda Orleańska-Pietras[1] *and Katarzyna Pogoda*[1*]

[1]Institute of Nuclear Physics Polish Academy of Sciences, PL-31342 Krakow, Poland

*Corresponding author: katarzyna.pogoda@ifj.edu.pl

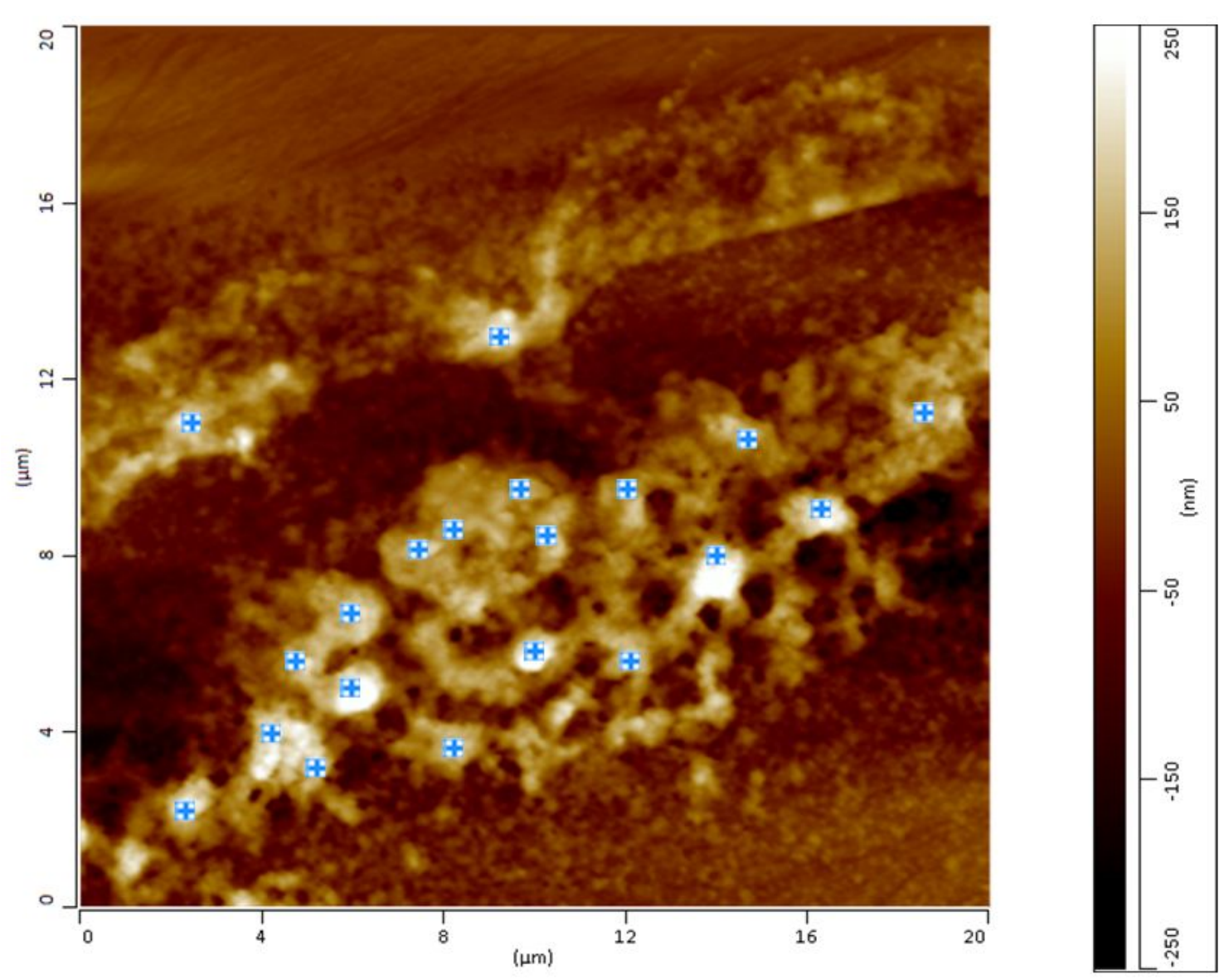

Figure. S1. Representative AFM topography of GPMVs isolated from U251 cells and deposited on a CaF_2 window, with the AFM-IR spectral acquisition positions indicated. Following drying, the GPMVs form a heterogeneous layer of membrane deposits rather than remaining as clearly

distinguishable individual vesicles. AFM-IR spectra were acquired at multiple locations across the deposited membrane material, as indicated by the markers.

Table S1. Spectral preprocessing workflows used for the different data analyses.

Analysis	Raman preprocessing	AFM-IR preprocessing
**Spectra shown in figures**	cosmic ray removal → Savitzky-Golay smoothing → baseline correction → vector normalization	background subtraction → Savitzky-Golay smoothing → vector normalization → second derivative
**Band integrals**	same as above	second derivative
**PCA**	cosmic ray removal → Savitzky-Golay smoothing → linear baseline correction → vector normalization	second derivative → linear baseline correction → vector normalization
**PLS-DA**	cosmic ray removal → constant offset elimination → linear baseline correction → multiplicative scatter correction → vector normalization	–

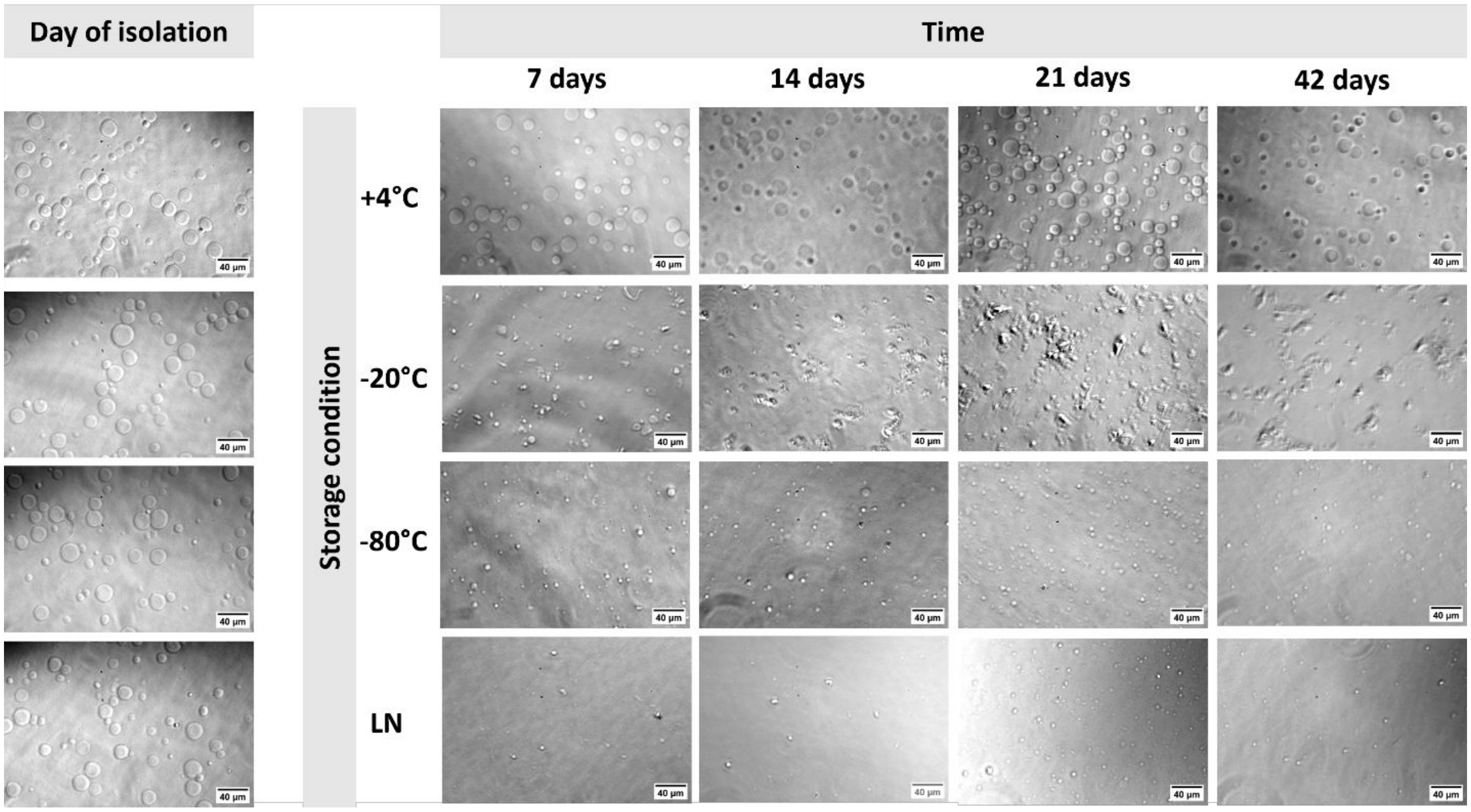

Figure S2. Optimalization of storage conditions for isolated GPMVs from LN229 cells (confluence 65%). Representative micrographs present morphology of vesicles, depending on the temperature of storage in time.

Table S2. Assignment of GPMVs Raman bands[1–5].

Raman band (cm^{1})	Assignment
543	Lipids, bending CH_2 in ring
579	Lipids, deformation C=O-C
606	Proteins
640, 646, 653	Proteins, tyrosine
664	Lipids, deformation C=O-C
701	Lipids, deformation of cholesterol ring
720	Lipids, phospholipids, symmetric stretching of choline group
750	Proteins, cytochrome c
758	Proteins, tryptophan

768	Proteins, tryptophan
779	Proteins, tryptophan
801	Proteins, tyrosine
813	Proteins, serine
828, 834	Proteins, tyrosine
843, 848	Proteins, tyrosine
853, 856	Proteins, tyrosine
864	Proteins, tyrosine
886	Proteins, tryptophan
935	Proteins N–$C_α$–C
942	Proteins N–$C_α$–C
956	Proteins N–$C_α$–C
979	Proteins, tryptophan, valine
1005	Proteins, phenylalanine
1020	Proteins, phenylalanine
1032, 1035	Proteins, phenylalanine
1055	Proteins, phenylalanine
1064	Lipids, stretching C-C
1090	Lipids, stretching C-C, phospholipids stretching P-O
1109	Lipids, stretching C-C
1130	Lipids, stretching C-C, proteins C-N, cytochromes c
1145	Proteins, C-N
1183	Lipids, stretching C-C
1206	Proteins, tyrosine
1215	Proteins, phenylalanine
1234	Proteins, amide III
1253	Proteins, amide III
1294	Proteins, amide III
1305	Lipids, twisting CH_2
1315	Proteins, cytochromes c
1340	Proteins, tryptophan, deformation $C_α$-H
1360	Proteins, serine, threonine
1378	Proteins, cytochrome c
1397	Lipids, bending CH_2
1405	Lipids, bending CH_2
1420	Lipids, bending CH_2
1440	Lipids, scissoring CH_2/CH_3; proteins deformation C-H
1460	Proteins deformation C-H; lipids bending CH_2/CH_3
1486	Proteins, deformation C-H

1496	Proteins, deformation C-H
1544	Proteins, indole ring (tryptophan)
1555	Proteins, indole ring (tryptophan)
1576	Proteins, tyrosine
1585	Proteins, cytochrome c
1605	Proteins, stretching NH_2, phenylalanine, tyrosine, tryptophan
1632	Proteins, amid I, phenylalanine, tyrosine, tryptophan
1650, 1660	Lipids stretching C=C; proteins, amid I
1672	Proteins, amide I
1694	Proteins, amide I
1730	Lipids, stretching C=O
1753	Lipids, stretching C=O
2853	Lipids symmetric stretching CH_2
2876	Proteins, lipids, asymmetric stretching CH_2
2933	Proteins, lipids, symmetric stretching CH_3
3060	Proteins, aromatic C-H

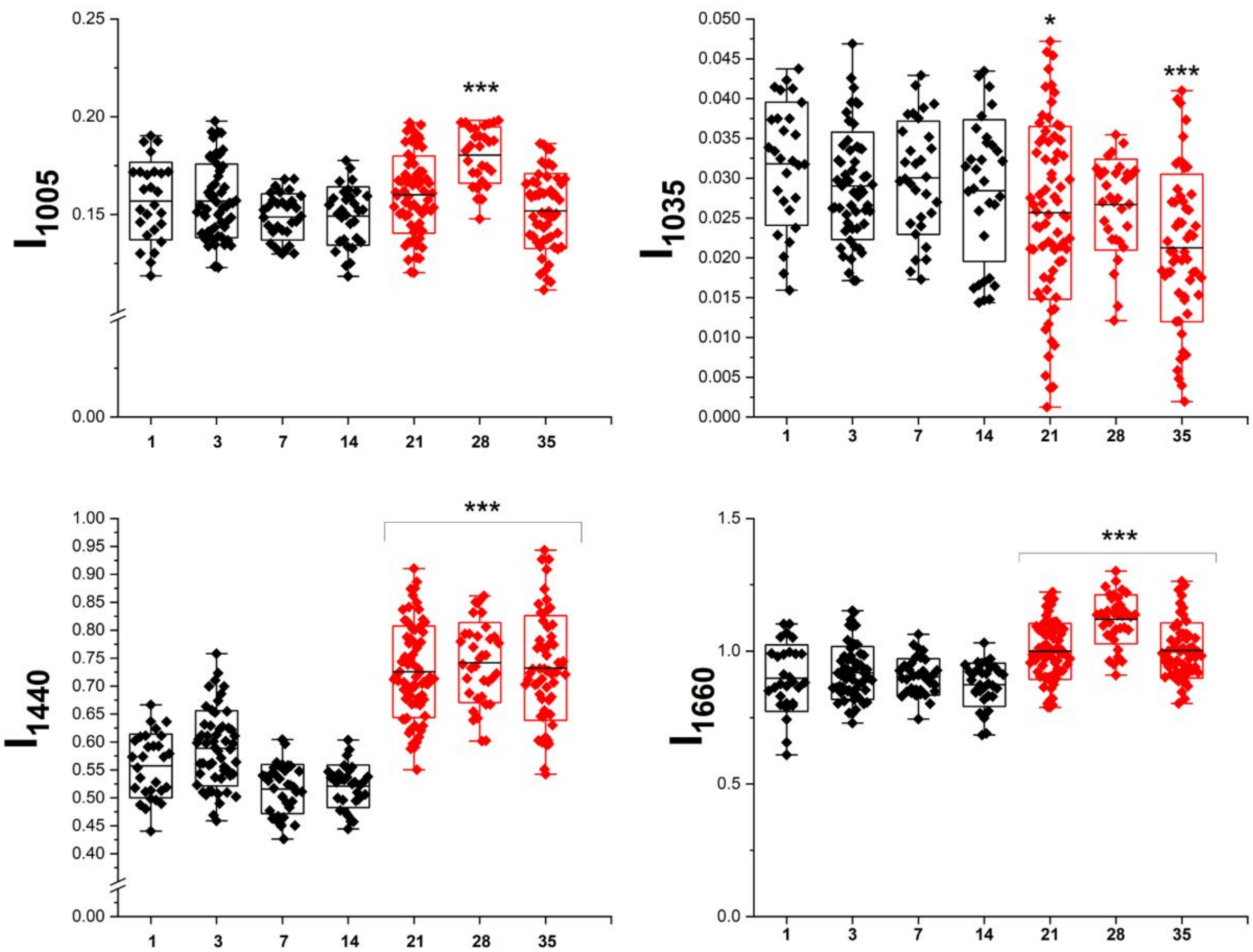

Figure S3. ANOVA analysis of integral intensities of the Raman bands: 1005, 1035, 1440, 1660 cm-1. *p < 0.05; **p < 0.01; ***; p < 0.001; data without * - no statistical significance versus day 1 (day of isolation).

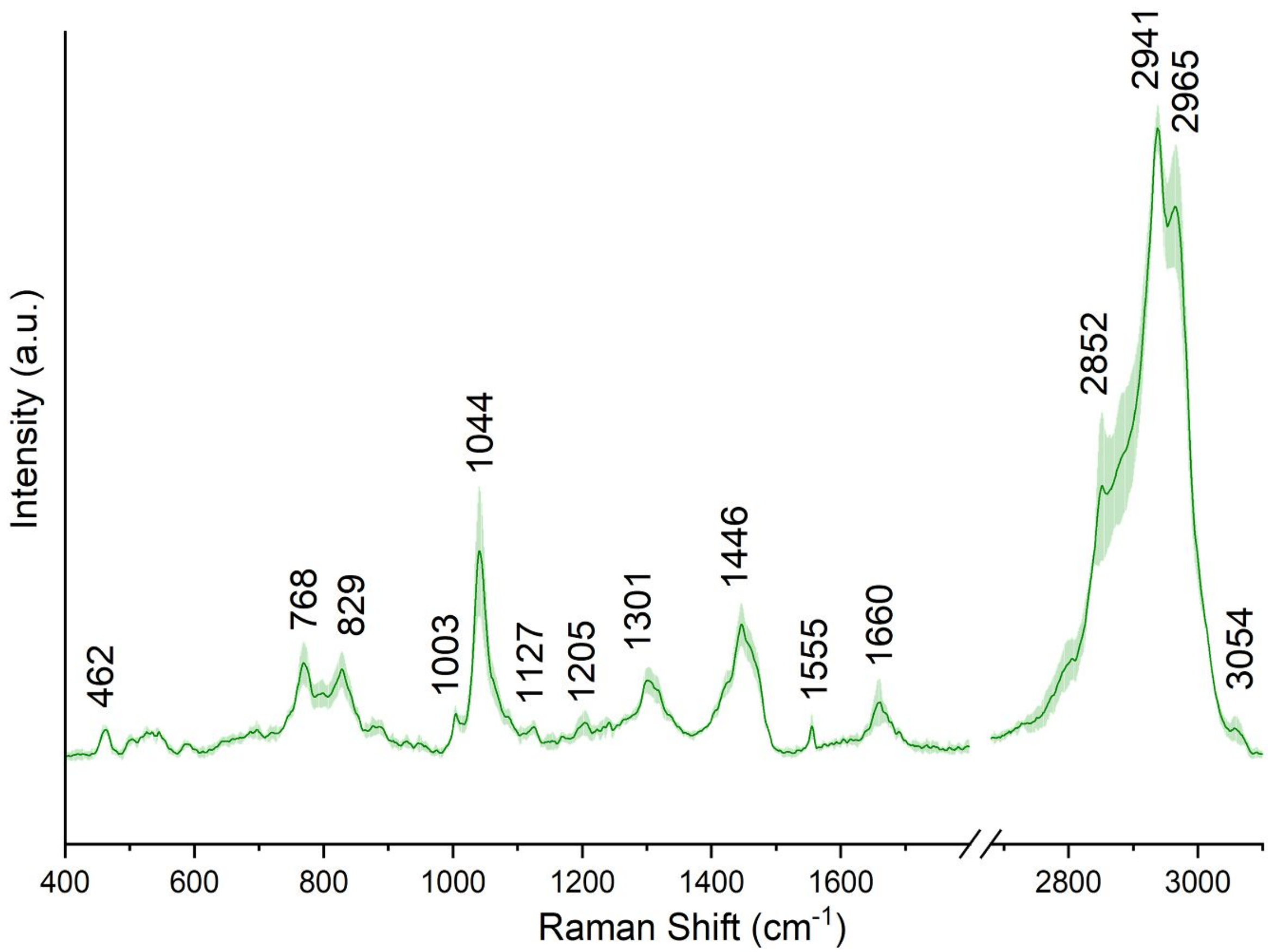

Figure S4. Average Raman spectrum with assignment of characteristic bands of dry, unrinsed isolation buffer crystals (laser line 532 nm).

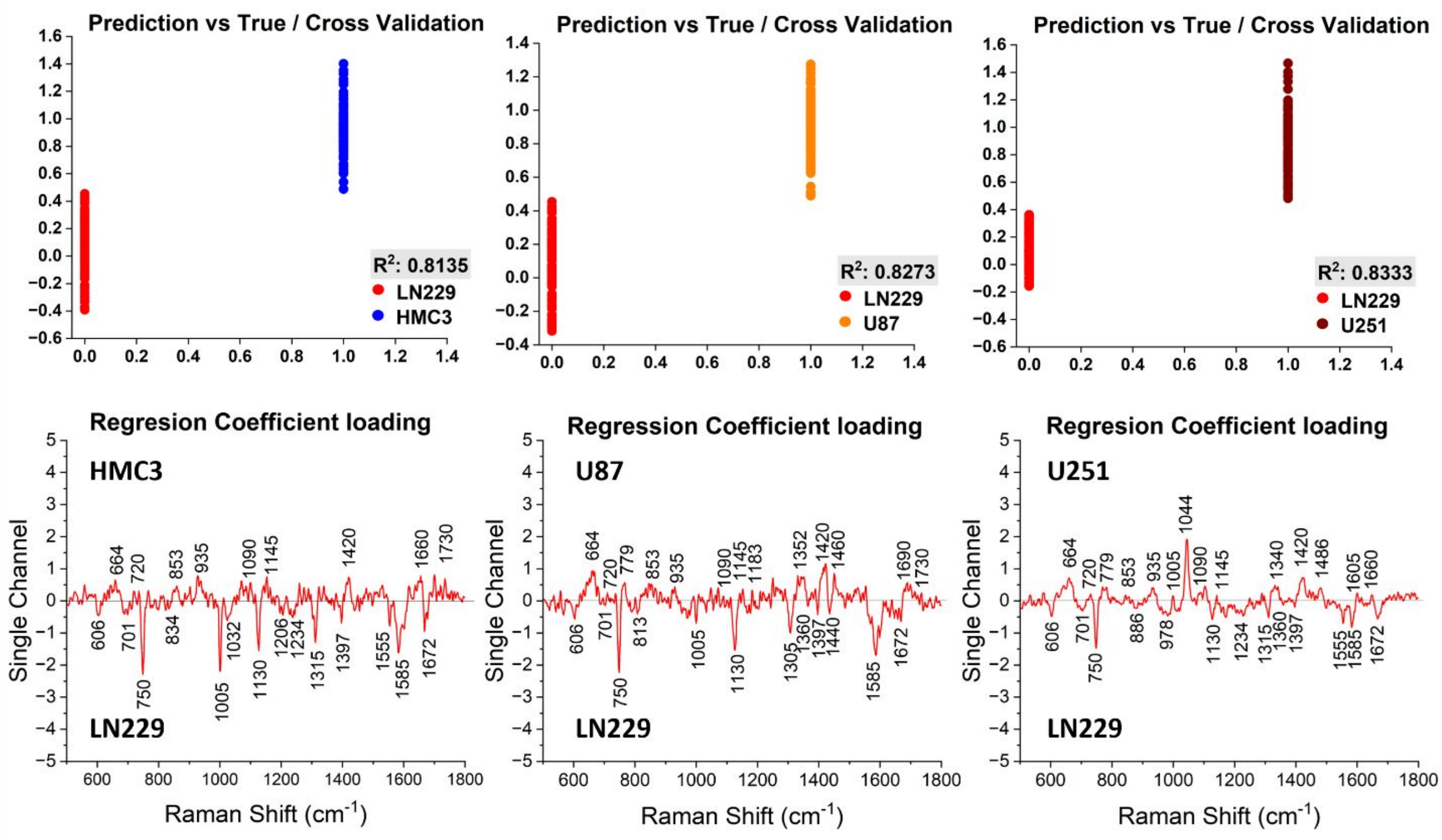

Figure S5. PLS-DA analysis of Raman spectra for LN229 vesicles versus HMC3, U87, U251. Spectral region 500-1800 cm^{-1}.

Table S3. Assignment of GPMVs IR bands[4,6–14].

Wavenumber (cm^{-1})	Assignment
**2958**	Lipids, proteins, asymmetric stretching CH_3
**2928**	Lipids, proteins, symmetric stretching CH_3
**2874**	Lipids, proteins, symmetric stretching CH_3
**2852**	Lipids, proteins, symmetric stretching CH_2, long chain of fatty acids
**1758**	Lipids, stretching C=O
**1744**	Lipids, cholesterol esters, stretching, C=O
**1706**	Lipids, deformation C=O
**1694**	Proteins, amide I, β-sheet in proteins, stretching C=O and deformation N-H
**1684**	Proteins, amide I; β-sheets in proteins, stretching C=O and deformation N-H

1674	Proteins, amide I
1664	Proteins, amide I, α-helices; stretching C=O and deformation N-H
1654	Proteins, amide I, α-helices in proteins, stretching C=O and deformation N-H
1642	Proteins, amide I, α-helices in proteins, stretching C=O and deformation N-H
1634	Proteins, amide I, β-sheet in proteins, stretching C=O and deformation N-H
1624	Proteins, amide I, β-sheet in proteins, stretching C=O and deformation N-H
1608	Proteins, amide I
1598	Proteins, amide I
1568	Proteins, amide II, in-plain deformation N-H and stretching C-N
1558	Proteins, amide II, in-plain deformation N-H and stretching C-N
1548	Proteins, amide II, in-plain deformation N-H and stretching C-N
1530	Proteins, amide II, in-plain deformation N-H and stretching C-N
1514	Proteins, tyrosine, stretching of the ring C=C
1476	Lipids, in-plain deformation CH_2
1466	Lipids, phospholipids, CH_2/CH_3
1456	Proteins, deformation CH_2, CH_3
1446	Proteins, deformation CH_2, CH_3
1438	Lipids, deformation CH_2, CH_3
1426	Lipids, phospholipids, CH_2/CH_3
1246	Lipids, phospholipids, asymmetric stretching PO_2^-
1232	Lipids, phospholipids, asymmetric stretching PO_2^-
1226	Lipids, sphingolipids, asymmetric stretching PO_2^-
1216	Lipids, sphingolipids, asymmetric stretching PO_2^-
1174	Lipids, cholesterol esters, fatty acids, stretching C-O
1154	Polysaccharides, asymmetric stretching CO-O-C
1134	Carbohydrates, asymmetric stretching C-O, C-C
1098	Carbohydrates, lipids, glycolipids, stretching C-O-C, C-C
1084	Lipids, sphingolipids, symmetric stretching PO_2^-
1068	Carbohydrates, lipids, glycolipids, stretching C-O-C, C-C
1054	Carbohydrates, glycolipids stretching C-O-C, C-C
1026	Lipids, sphingolipids, symmetric stretching C-O
1010	Carbohydrates, stretching C-O, C-C, C-OH in-plain bending
984	Carbohydrates, stretching C-O
790	Carbohydrates, stretching C-O-C

**780**	Carbohydrates, stretching C-C , C-H in-plain bending

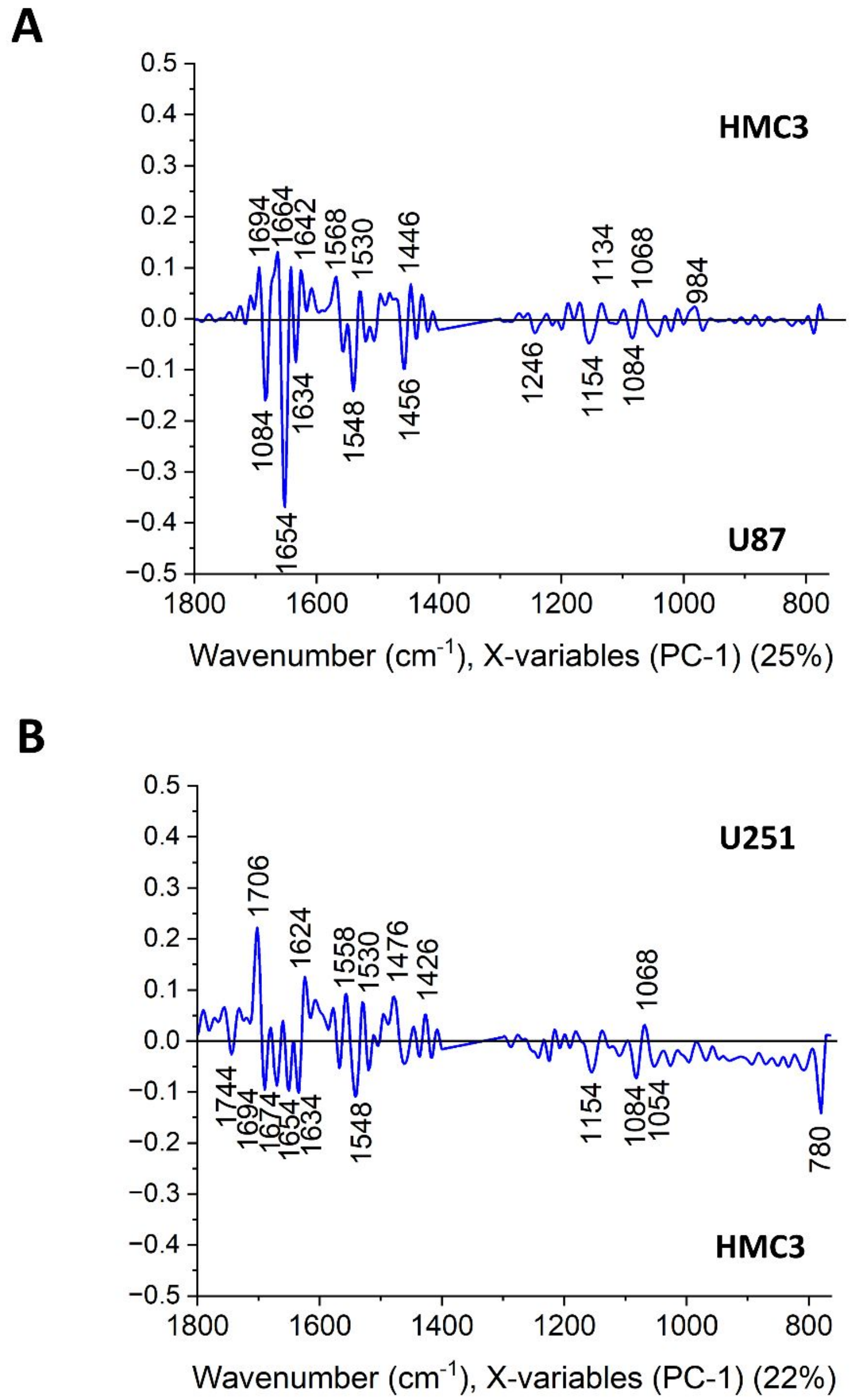

Figure S6. PC1 loading plots from pairwise PCA of second-derivative AFM-IR spectra of GPMVs. (A) HMC3 versus U87 (PC1, 25% of the total variance) and (B) HMC3 versus U251 (PC1, 22%

of the total variance). The analysis was performed in the spectral ranges of 1800–1400 and 1300–764 cm⁻¹.

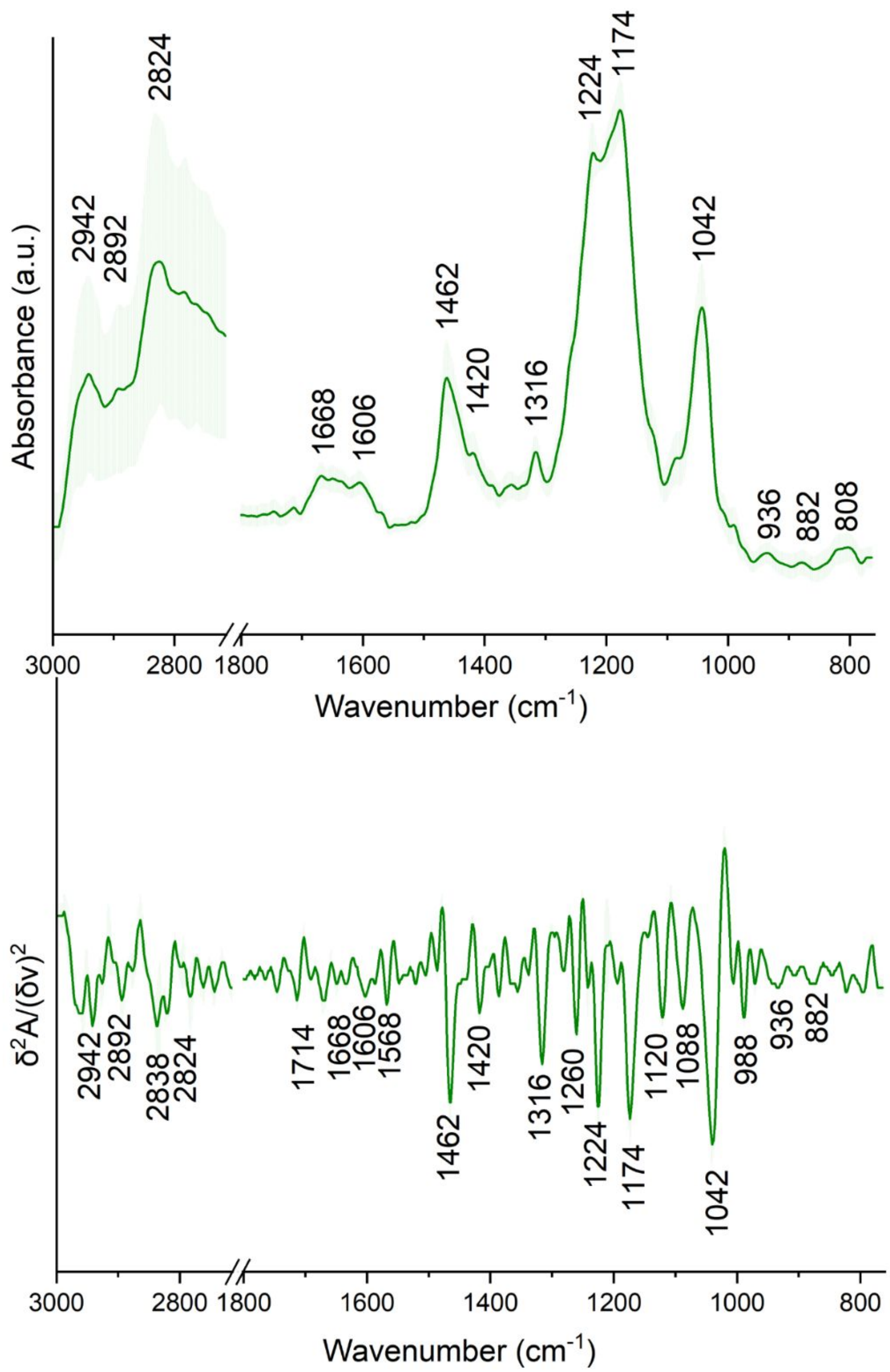

Figure S7. Average AFM-IR spectrum (top) and second-derivative spectrum (bottom) with highlighted characteristic bands of dry, unrinsed isolation buffer crystals.

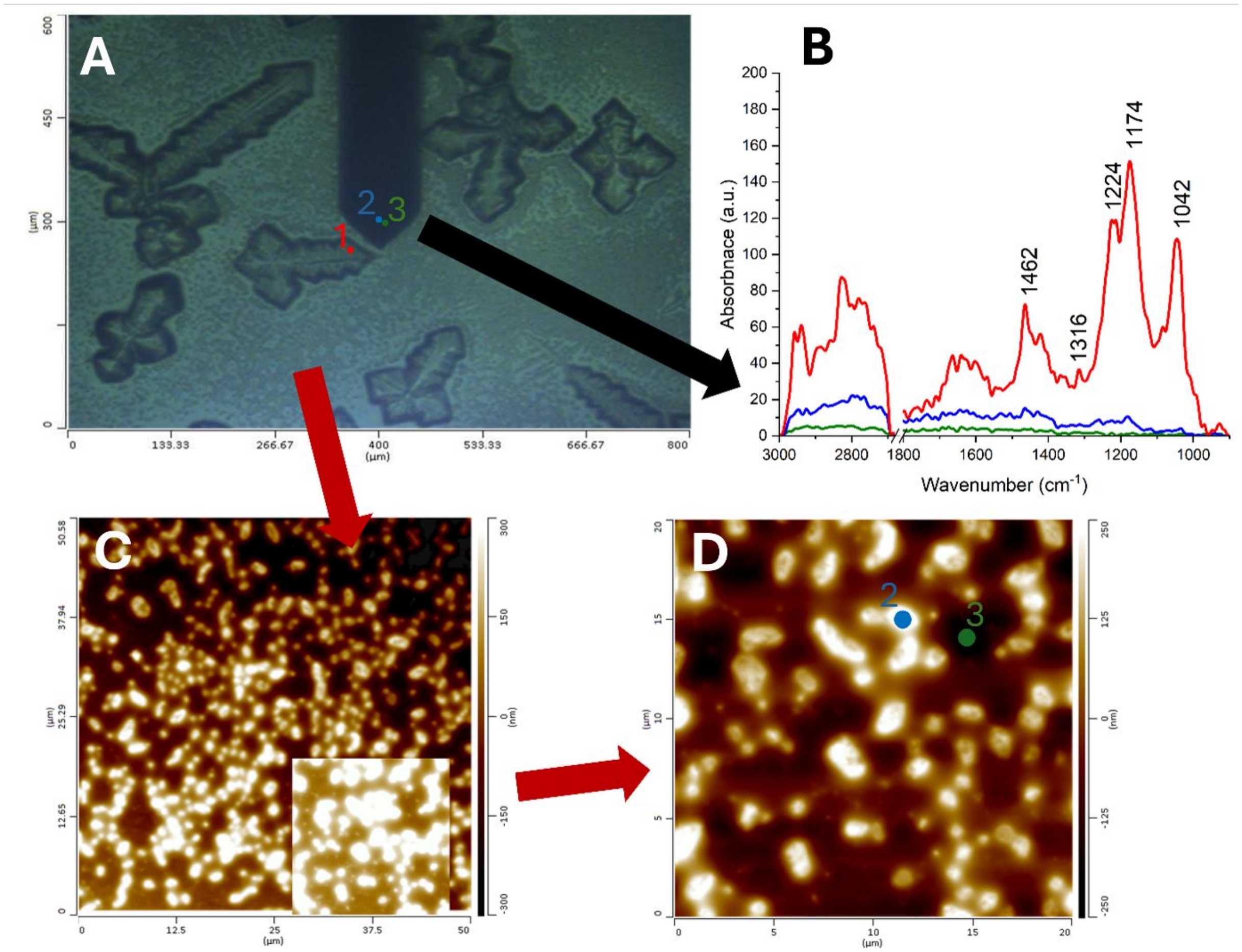

Figure S8. Optical micrograph (A) and AFM topographies (C, D) of a dried, unrinsed isolation-buffer sample deposited on a CaF_2 window, together with representative AFM-IR spectra (B) collected at the indicated locations. The spectra correspond to measurements performed directly on a large buffer crystal (red spectra, position 1), on a small buffer crystal visible in the AFM topography (blue spectra, position 2), and in a region between visible crystals (green spectra, position 3).

References

(1) Czamara, K.; Majzner, K.; Pacia, M. Z.; Kochan, K.; Kaczor, A.; Baranska, M. Raman Spectroscopy of Lipids: A Review. *J. Raman Spectrosc.* **2015**, *46* (1), 4–20. https://doi.org/https://doi.org/10.1002/jrs.4607.

(2) Nowakowska, A. M.; Pieczara, A.; Korona, W.; Borek-Dorosz, A.; Adamczyk, A.; Dawiec, P.; Leszczenko, P.; Orleanska, J.; Machalska, E.; Orzechowska, B.; Orzechowska, S.; Brzozowski, K.; Krynicka, W.; Majzner, K.; Malek, K.; Baranska, M. The Raman Map of the Human Cell. *Anal. Chem.* **2025**, *97* (30), 16374–16382. https://doi.org/10.1021/acs.analchem.5c02035.

(3) Rygula, A.; Majzner, K.; Marzec, K. M.; Kaczor, A.; Pilarczyk, M.; Baranska, M. Raman Spectroscopy of Proteins: A Review. *J. Raman Spectrosc.* **2013**, *44* (8), 1061–1076. https://doi.org/https://doi.org/10.1002/jrs.4335.

(4) Chrabąszcz, K. Spectroscopic Active Probes for Investigation of Lipid Transformation in Cells and Membranes. *J. Phys. Chem. B* **2026**, *130* (6), 1743–1754. https://doi.org/10.1021/acs.jpcb.5c05981.

(5) Xu, J.; Karl J, M. Raman Micro-Spectroscopy as a Tool to Study Immunometabolism. *Biochem. Soc. Trans.* **2024**, *52* (2), 733–745.

(6) Chrabąszcz, K.; Pogoda, K.; Cieżak, K.; Panek, A.; Kwiatek, W. M. Sensing Biomolecules Associated with Cells' Radiosusceptibility by Advanced Micro- and Nanospectroscopy Techniques. *ACS Sensors* **2024**, *9* (9), 4887–4897. https://doi.org/10.1021/acssensors.4c01455.

(7) Pragnąca, A.; Antolak, A.; Krysiak, Z. J.; Leśniak, M.; Borkowska, A.; Zdanowski, R.; Malek, K. Marker-Independent Vibrational Spectroscopy Imaging Recognizes the Hypoxia Effect in the Human Brain Endothelium. *Sci. Rep.* **2025**, *15* (1), 26112. https://doi.org/10.1038/s41598-025-11000-2.

(8) Kong, D.; Wenyu, P.; Zong, R.; Cui, G.; Yu, X. Morphological and Biochemical Properties of Human Astrocytes, Microglia, Glioma, and Glioblastoma Cells Using Fourier Transform Infrared Spectroscopy. *Med. Sci. Monit.* **2020**, *26*, e925754-1. https://doi.org/10.12659/MSM.925754.

(9) Sandt, C.; Nadaradjane, C.; Richards, R.; Dumas, P.; Sée, V. Use of Infrared Microspectroscopy to Elucidate a Specific Chemical Signature Associated with Hypoxia Levels Found in Glioblastoma. *Analyst* **2016**, *141* (3), 870–883.

https://doi.org/10.1039/C5AN02112J.

(10) Wiercigroch, E.; Szafraniec, E.; Czamara, K.; Pacia, M. Z.; Majzner, K.; Kochan, K.; Kaczor, A.; Baranska, M.; Malek, K. Raman and Infrared Spectroscopy of Carbohydrates: A Review. *Spectrochim. Acta Part A Mol. Biomol. Spectrosc.* **2017**, *185*, 317–335. https://doi.org/https://doi.org/10.1016/j.saa.2017.05.045.

(11) Al-Kelani, M.; Buthelezi, N. Advancements in Medical Research: Exploring Fourier Transform Infrared (FTIR) Spectroscopy for Tissue, Cell, and Hair Sample Analysis. *Ski. Res. Technol.* **2024**, *30* (6), e13733. https://doi.org/https://doi.org/10.1111/srt.13733.

(12) Gałat, A. Study of the Raman Scattering and Infrared Absorption Spectra of Branched Polysaccharides. *Acta Biochim. Pol.* **1980**, *27* (2), 135–142.

(13) Holcombe, B.; Foes, A.; Banerjee, S.; Yeh, K.; Wang, S.-H. J.; Bhargava, R.; Ghosh, A. Intermediate Antiparallel β Structure in Amyloid β Plaques Revealed by Infrared Spectroscopic Imaging. *ACS Chem. Neurosci.* **2023**, *14* (20), 3794–3803. https://doi.org/10.1021/acschemneuro.3c00400.

(14) Antolak, A.; Pragnaca, A.; Augustyniak, K.; Zahra, S. T.; Pięta, Ł.; Wislocka-Orlowska, A.; Majzner, K.; Baranska, M.; Malek, K. IR Map of the Human Cell. *Anal. Chem.* **2026**. https://doi.org/10.1021/acs.analchem.6c00513.
